# Artificial sweeteners stimulate horizontal transfer of extracellular antibiotic resistance genes through natural transformation

**DOI:** 10.1038/s41396-021-01095-6

**Published:** 2021-09-01

**Authors:** Zhigang Yu, Yue Wang, Ian R. Henderson, Jianhua Guo

**Affiliations:** 1 Advanced Water Management Centre, The University of Queensland, St. Lucia, Brisbane, QLD 4072 Australia; 2 Institute for Molecular Bioscience, The University of Queensland, St. Lucia, Brisbane, QLD 4072 Australia

**Keywords:** Antibiotics, Public health

## Abstract

Antimicrobial resistance has emerged as a global threat to human health. Natural transformation is an important pathway for horizontal gene transfer, which facilitates the dissemination of antibiotic resistance genes (ARGs) among bacteria. Although it is suspected that artificial sweeteners could exert antimicrobial effects, little is known whether artificial sweeteners would also affect horizontal transfer of ARGs via transformation. Here we demonstrate that four commonly used artificial sweeteners (saccharin, sucralose, aspartame, and acesulfame potassium) promote transfer of ARGs via natural transformation in *Acinetobacter baylyi* ADP1, a model organism for studying competence and transformation. Such phenomenon was also found in a Gram-positive human pathogen *Bacillus subtilis* and mice faecal microbiome. We reveal that exposure to these sweeteners increases cell envelope permeability and results in an upregulation of genes encoding DNA uptake and translocation (Com) machinery. In addition, we find that artificial sweeteners induce an increase in plasmid persistence in transformants. We propose a mathematical model established to predict the long-term effects on transformation dynamics under exposure to these sweeteners. Collectively, our findings offer insights into natural transformation promoted by artificial sweeteners and highlight the need to evaluate these environmental contaminants for their antibiotic-like side effects.

## Introduction

Antimicrobial resistance (AMR) has been recognized as a global public health challenge [1] that is currently responsible for over 700,000 deaths annually [2]. Estimates of annual death will be increased to 10 million, if no action is being taken now [3]. Bacteria can acquire antibiotic resistance through the selection of spontaneous mutations that confer resistance to particular drugs, or through horizontal gene transfer (HGT), in which conjugation, transduction and transformation are the three principal pathways [4, 5]. Conjugation and transduction require specific apparatuses (conjugative pili and phage virions, respectively) for the transfer of antibiotic resistance genes (ARGs) between bacterial cells. Differently, transformation involves the uptake, integration and functional expression of extracellular DNA by naturally competent bacteria [6, 7].

Antibiotics are well recognized as a selective pressure increasing spread of ARGs and selecting for ARG-containing bacteria, which in turn drives AMR evolution of bacteria [8–10]. Antibiotics induce stresses on bacteria via targeting on bacterial cellular components with their functional groups. Artificial sweeteners, substitutes of table sugar in foods and beverage, contain antibiotic-like groups. For instance, saccharin and acesulfame potassium contain a sulfonyl group, which is similar to the functional group of the sulphonamide antibiotics (Table S1). As well, these sweeteners are also reported to exert antimicrobial effects [11] and can induce antibiotic-like shift in gut microbiota [12]. Accordingly, we hypothesized that these widely-used food additives [13–15] might also contribute to the dissemination of antibiotic resistance among bacteria. Our recent study has demonstrated that the widely-used artificial sweeteners can promote horizontal transfer of ARGs via conjugation [16]. These sweeteners promoted pili formation and increased expression of conjugative transfer-related genes located on the plasmid. However, it remains untested whether these sweeteners could promote ARG transfer via transformation. Specifically, little is known about whether the sweeteners could enhance the competence of bacterial cells or shape plasmid structure, or both during transformation process.

In Gram-negative bacteria, natural transformation is dependent on bacteria producing a DNA uptake apparatus (Type IV pili [T4P]) and DNA translocation machinery (Com system) [17, 18]. The T4P contacts extracellular DNA and mediates translocation across the outer membrane into the periplasmic space. Once in the periplasm the Com system engages with the DNA, converting it to a single strand that can be translocated across the inner membrane before being integrated into the host genome. Such transformability can diversify bacterial genome and benefit the host with rapid adaptation and niche expansion. Moreover, in the natural environment, extracellular DNA molecules that carry ARGs are secreted by living cells or released by cell lysis; they are abundant and persistent over weeks [19, 20]. These free DNA molecules can be a source and vehicles of ARGs transfer in microbial populations and play critical roles in microbial ecology and evolution. Unlike some conjugative plasmids that contain active partitioning systems and can be persist in the hosts, non-mobile plasmids can be easily lost during cell proliferation, under non-selective conditions [21]. Until now, little is known whether the sweeteners could affect the persistence of the acquired plasmid in the recipient. Given the antimicrobial effects of artificial sweeteners, we hypothesize that the sweeteners could promote plasmid persistence in the recipient. This will suggest the critical roles of artificial sweeteners in the modulation of microbial evolution.

In this work, we established five model systems for transformation and investigated the capacity of four commonly used artificial sweeteners (saccharin (SAC), sucralose (SUC), aspartame (ASP), and acesulfame potassium (ACE-K)) to promote uptake of ARGs. The mechanism underlying the increased transformation was uncovered by a series of analyses revealing changes in cell membrane permeability, and the expression of genes and proteins relevant to the transformation process (competence). The maintenance of ARGs was also assessed under exposure to these sweeteners. Moreover, the long-term effect of artificial sweeteners on transformation dynamics was predicted through a mathematical model. To the best of our knowledge, this is the first study to explore how artificial sweeteners promote the horizontal transfer of multiple ARGs via natural transformation. These findings provide important evidence that artificial sweeteners promote the spread of ARGs and highlight the potential effect on microbial ecology and evolution, as well as health risks associated with increasing use of such food additives.

## Methods

### Bacteria strains and artificial sweeteners

*A. baylyi* ADP1, a naturally competent strain [22–24], and a mutant *∆comFEBC* strain (*lifO-lipB::aphA3*′*ΔcomFEBC::DHFR-1* [25]) were used as the recipient in this study. A non-conjugative plasmid, pWH1266, that contains ampicillin (Amp) and tetracycline (Tet) resistance genes (*bla*_*TEM-1*_ and *tetA*), was used as the source of extracellular DNA for transformation experiments. Four analytic reagents of artificial sweeteners including SAC, SUC, ASP and ACE-K (detailed information shown in Table S1), were obtained from Sigma-Aldrich (USA) and were dissolved in Milli-Q water as stock solutions for further use.

### Determining inhibitory concentrations of artificial sweeteners or antibiotics

The concentrations of four sweeteners and two antibiotics corresponding to 90% growth inhibition (IC90) of *A. baylyi ADP1* were determined by plate-reader (Tecan Infinite M200, Swiss) measurements. Typically, *A. baylyi* ADP1 was cultured overnight in LB media. Cell concentrations were adjusted to ~10^5^ CFU/mL before being transferred to 96-well plates. Each well contained 75 µL cell suspension and 75 µL LB media with different concentrations of artificial sweeteners or antibiotics (Amp and Tet). After incubation at 30 °C for 20 h, the plates were scanned by the plate reader at a wavelength of 600 nm and the optical density (OD_600_) of each well was recorded to calculate IC90 values. Each experiment was run in triplicates. Simultaneously, the control was set up by adding Milli-Q water or LB broth instead of any chemicals.

### Transformation model setup

*A. baylyi* ADP1 was inoculated in LB media without antibiotics. After overnight culture, 1% of cell suspension was transferred to fresh LB media and was cultivated on a shaker at 30 °C, 130 rpm until reaching exponential growth phase. Cells were then collected by centrifuging at 5000 rpm (Eppendorf 5810R, Germany) for 5 min and was washed with sterilized phosphate-buffered saline (PBS) twice to remove any LB residues. The collected cell was resuspended and the OD_600_ value of cell suspension was adjusted to about 1.1 with PBS. The pWH1266 plasmid was always freshly prepared and suspended in elution buffer (10 mM Tris-HCl), and was added to the cell suspension, to a final concentration of 0.8 ng/µL plasmid (equals to 8.3 ×10^7^ copies/µL). Since energy is needed for bacteria to uptake the external DNA fragment, carbon source was provided as the carbon level in the effluent of wastewater treatment plant. Here, sodium acetate (50 mg/L) was added to the mixture of bacterial and plasmid. The mixture was then exposed to various concentrations (0, 0.03, 0.3, 3, 30, 60 and 300 mg/L) of four artificial sweeteners (Model I). After that, the transformation system was well mixed and incubated at room temperature for 6 h (Text S1; Fig. S1). Total cell numbers were calculated by plating the samples on agar containing no antibiotics, while the number of transformants were estimated by enumerating colonies after growth on agar containing 100 mg/L Amp and 20 mg/L Tet. The transformation frequency was calculated as the ratio between the transformants number and the total cell number.

Meanwhile, effects of sucrose and glucose, which are commonly used sugars, were also investigated by following the same procedures. Models II and III refer to the pre-exposure of plasmid (pre-exposure 1) and the recipient (pre-exposure 2) to artificial sweeteners, respectively. Detailed methods are described in Text S2. In addition, we tested with a Gram-positive pathogen *Bacillus subtilis* (ATCC 6051) [26] that resides in human gastrointestinal tract (Model IV). The pWH1266 plasmid was used as extracellular DNA, and the assay followed the same procedure as described above. To further validate the phenomenon at a community level, an in vitro transformation model (Model V) was also established by using mice faecal bacteria as the recipient and a *gfp*-tagged pKJK5 plasmid as extracellular DNA. Once successful transferred to a faecal bacterium, *gfp* can be expressed and produce green-fluorescent cells. Based on such fluorescent reporter, faecal bacteria that successfully receive pKJK5 plasmid (becoming transformants) can be sorted by a fluorescence-activated cell sorting (FACS) technique (Text S3; Fig. S2) [27]. Typically, transformants were sorted based on triple gates: the gate of forward scatter-H vs side scatter-H plot for bacterial size; the gate of forward scatter-H vs forward scatter-W plot for singlet; the third gate of 561_TexaRed-A vs 488_SYBR-A plot for green-fluorescent cells. Detection results were analyzed by FlowJo 7.6.

### Measurement of cell membrane permeability and growth curves

Cell membrane permeability of *A. baylyi* ADP1 exposed to various concentrations (0, 0.03, 0.3, 3, 30, 60 and 300 mg/L) of artificial sweeteners and sugars (sucrose and glucose) were measured by a CytoFLEX S flow cytometer (Beckman Coulter, USA). Propidium iodide (PI) dye was used for measurement of cell membrane permeability. The positive binding group using heated cells was confirmed with the control without sweeteners treatment (Fig. S3) [28]. The growth curves of *A. baylyi* ADP1 exposed to artificial sweeteners were also examined. Details of methods were described in Texts S4 and S5.

### Plasmid extraction and detection of resistance genes by PCR assays

To confirm whether pWH1266 was successfully taken up by *A. baylyi* ADP1, we extracted plasmids from those transformants and conducted PCR assays of two ARGs (*bla*_*TEM-1*_ and *tetA*) carried by the pWH1266 plasmid (Text S6; Table S2). Visualization of all bands were conducted by SYBR safe DNA gel staining and the GeneRuler 1 kb DNA ladder.

### RNA extraction, genome-wide RNA sequencing and transcriptomic analysis

Transcriptomic responses of *A. baylyi* ADP1 to artificial sweeteners were investigated by the whole genome-wide RNA sequencing. Cell suspensions treated with 30 mg/L artificial sweeteners were the treated group, while those without artificial sweeteners treatment were the control group. After 2-h treatment, cell pellets were collected by centrifuging at 4 °C, 6000 rpm for 6 min. The total RNA was then extracted using the RNeasy Mini Kit (QIAGEN, Germany) according to the manufacturer’s instructions, with an extra cell lyse step of beads-beating. The extracted RNA samples were stored in −80 °C freezer and were shipped in dry ice to Novogene (Hong Kong SAR, China). Construction of strand-specific cDNA library and high-throughput sequencing of paired end genome were conducted for transcriptomic analysis. The reference genome of *A. baylyi* ADP1 (NC_005966.1) from National Centre for Biotechnology Information (NCBI) was used as the alignment database. All samples were prepared in triplicate. Fragments per kilobase of a gene per million mapped reads was measured to quantify gene expression. Differences of gene expression between the control (without addition of artificial sweeteners) and the sweetener-treated groups were presented as log_2_ fold-changes (LFC) [29]. Correspondingly, the absolute fold changes of relevant genes expression were calculated and were presented in this work. Significance in transcriptome data for expression of genes was determined by a false discovery rate (FDR)-adjusted *p* value (*p*_*-adj*_) less than 0.05.

### Proteomic sequencing and data analysis

After a 6-h exposure to 30 mg/L of artificial sweeteners, cells were collected for protein extraction. The total bacterial proteins were digested in S-Trap filters (ProtiFi, Huntington, USA) by following the manufacturer’s protocol and a previous study [30]. The detailed protocol and data analysis were described in Text S7. Raw sequencing data were processed by a Thermo Proteome Discoverer (version 2.2.0.388) towards the database of *A. baylyi* (strain ATCC3305/BD413/ADP1). Abundance ratios of each protein between the sweetener-treated groups and the control group were calculated. A stringency cut-off of false discovery rate (FDR, *q* value) less than 0.01 was applied to identify the proteins with significant different expression levels.

### Plasmid persistence assay

To examine the persistence of the acquired plasmid in bacterial cells, persistence assays for pWH1266 in transformants were conducted in LB media containing 3 mg/L of artificial sweetener. Every 24 h, 1% of cell suspension was transferred into the next tube with fresh LB media and artificial sweeteners. This was repeated for five subculture cycles. After each cycle, the fraction of plasmid-bearing cells was calculated by dividing the number of plasmid-bearing populations with the total colonies number. Detailed information is provided in Text S8.

The fitness cost of the acquired pWH1266 plasmid was also calculated during 5-day assay, according to the previous method [31]. The competition index (CI) was daily calculated by dividing the ratio of resistant (plasmid-maintaining) and susceptible (plasmid-free) numbers. Afterwards, a linear regression model (*s* = ln (CI)/*t*) was plotted to obtain the selection coefficient *s*, shown as the slope. The relative fitness (*w*) was then determined with the equation *w* = 1 + *s*.

### Prediction of cumulative effects on transformation

In this study, the long-term effect of artificial sweeteners on natural transformation was also predicted with an ordinary differential equation (ODE) model [29], which involves in the dynamic shift between the recipient, transformant and free plasmid. The number of the recipient and transformation cells, as well as the free plasmid copy number were calculated based on the following equations:

1 $$\frac{{dN_0}}{{dt}} = r_0N_0\left( {1 - \frac{{N_0 + N_1}}{K}} \right) - d_0N_0 - \mu PN_0$$

2 $$\frac{{dN_1}}{{dt}} = r_1N_1\left( {1 - \frac{{N_0 + N_1}}{K}} \right) - d_1N_1 + \mu PN_0$$

3 $$\frac{{dP}}{{dt}} = - \mu PN_0 + d_1N_1\lambda - \theta P$$

The model was implicitly calibrated with two key variables (transformation frequency *μ* and death rate *d*) as follows:

4 $$\min LS\left( {\mu ,d} \right) = \alpha \cdot \left[ {N_{0,obs}\left( 6 \right) - N_{0,sim}\left( 6 \right)} \right]^2 + \beta \cdot \left[ {N_{1,obs}\left( 6 \right) - N_{1,sim}\left( 6 \right)} \right]^2$$

5 $$\left\{ {\begin{array}{*{20}{c}} {\mu = K_\mu \cdot \mu _{ref}} \\ {d = K_d \cdot d_{ref}} \end{array}} \right.$$

Where *N*_0,obs_ (6) and *N*_1,obs_ (6) are the lab-obtained numbers of the recipient and transformants after 6 h. *N*_0,sim_ (6) and *N*_1,sim_ (6) are the predicted numbers of the recipient and transformants. Explicit constraints of two key variables were set up and the detailed information was shown in the Text S9 and Tables S3–S9. All parameters were calculated or measured based on the phenotypic results of transformation at 30 mg/L of artificial sweeteners. In addition to assess the long-term effect of each sweetener, the combined effect of four sweeteners was also evaluated. Based on the hypothesis that all of the recipient cells become transformant in the long run, the stability time that here is defined as the time for *N*_*1*_ reaching 95% of the final *K* value was calculated. In addition, the individual amplify factor of *Kμ* (scale factor of transformation frequency) or *Kd* (scale factor of death rate) in each sweetener-treated group was used to calculate the combined amplify factor. All parameters used in the models are shown in Table 1.

**Table 1:** Parameters involved in ODE model and implicit calibration model.

Parameter	Description	Value
*N*_*0*_	The number of wild type *A. baylyi*	
*N*_*1*_	The number of transformant (*A. baylyi* with pWH1266)	
*P*	Free plasmid copies	
*K*	Environmental carrying capacity, CFU/mL	3 × 10^8^
*t*	Time (h)	
*r*_*0*_	Growth rate of wild type *A. baylyi*	0.2
*r*_*1*_	Growth rate of transformant	0.2
*d*_*0*_	Death rate of wild type *A. baylyi*	
*d*_*1*_	Death rate of transformant	
*μ*	Transformation frequency	
*λ*	Copy number of plasmid in transformant	1000
*θ*	Decay rate of plasmid in environment	0.2
*LS*	Objective function of the two-parameter model, indicating the deviation between simulated and observed values in ODE model	
*α*	Weight factor of the error between simulation and observation values of *N*_*0*_	
*β*	Weight factor of the error between simulation and observation values of *N*_*t*_	
*μ*_*ref*_	Reference value of transformation frequency	10^−8^
*d*_*ref*_	Reference value of death rate	0.11
*K*_*μ*_	Scale factor of transformation frequency	
*K*_*d*_	Scale factor of death rate	
*K*_μ_*	Optimal scale factor of transformation frequency	
*K*_d_*	Optimal scale factor of death rate	

### Statistical analysis

All the experiments were conducted independently at least in biological triplicate. All data were expressed as mean ± SD and were analysed with SPSS 27.0 (SPSS, Chicago, USA). The phenotypic results were analysed by Analysis of variance (ANOVA) and Independent-sample *t* test method, with the Benjamini-Hochberg correction [32]: *p* values less than 0.05 are considered to be statistically significant.

## Results

### Artificial sweeteners alter transformation frequency

To test whether artificial sweeteners affect transformation, a DNA uptake experiment was set up by exposing *Acinetobacter baylyi* ADP1 to four common artificial sweeteners (SAC, SUC, ASP, and ACE-K) for 6 h (Fig. S1) in the presence of non-mobile pWH1266 plasmid (carrying ARGs such as *bla*_*TEM-1*_ and *tetA*). The concentrations of artificial sweeteners chosen (0, 0.03, 0.3, 3, 30, 60 and 300 mg/L) were environmentally- and clinically-relevant [33, 34], and were determined to be below the IC90 for *A. baylyi* ADP1 (Fig. S4). The growth of *A. baylyi* ADP1 can be arrested by artificial sweeteners and especially such growth arrest can be enhanced at higher concentration (i.e., 300 mg/L) of the sweeteners (Fig. S5; Table S10).

**Fig. 1:**
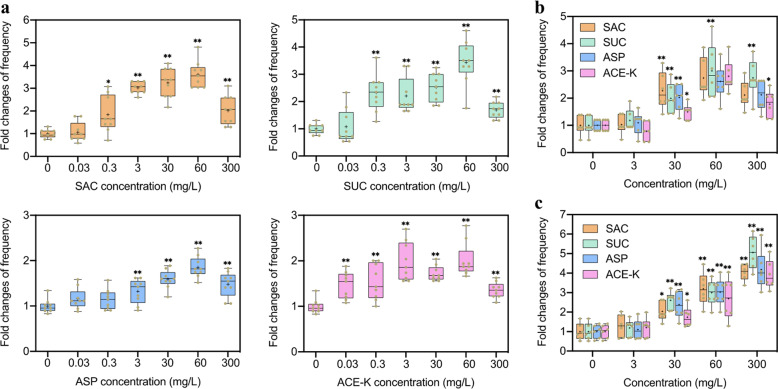
Effects of artificial sweeteners (SAC, SUC, ASP, and ACE-K) on the transformation frequency of extracellular ARGs to competent cells. **a** Fold changes of transformation frequency of pWH1266 plasmid in *A. baylyi* ADP1 under different concentrations of artificial sweeteners. At high concentrations (> 0.3 mg/L), artificial sweeteners promote the transformation (*N* = 9; ANOVA, *p* < 0.05). **b** Fold changes of transformation frequency of pWH1266 plasmid in *Bacillus subtilis* under exposure to different concentrations of artificial sw0eeteners (*N* = 6). **c** Fold changes of transformation frequency of *gfp*-encoded pKJK5 plasmid in mice faecal bacteria under exposure to different concentrations of artificial sweeteners (*N* = 6). Significant differences between individual sweetener-treated groups and the control (0 mg/L of sweeteners) were tested with Independent-sample *t* test, *p < 0.05 and **p < 0.01.

When compared to the control, the transformation frequencies showed a concentration-dependent pattern of increase in the presence of artificial sweeteners, with the largest increase of 3.6- (*p* = 5.0 × 10^−10^), 3.4- (*p* = 1.9 × 10^−8^), 1.9- (*p* = 6.0 × 10^−10^), and 2.0-fold (*p* = 6.0 × 10^−10^) induced by these sweeteners at 60 mg/L, respectively (Fig. 1a). This increase was associated with increased number of colonies on the selective transformant plates and with insignificant changes in the total number of viable cells in the population (Figs. S6 and S7). By contrast, sucrose and glucose only induced a lower fold increase throughout at 60 mg/L, even if they could promote the transformation frequency (Fig. S8). For example, at 60 mg/L, there are approximately two times less of transformation frequency induced by both sugars, compared to SUC (*p* = 0.00008).

To test whether the damage to the DNA by the artificial sweeteners might enhance transformation, we pre-exposed plasmid pWH1266 to 300 mg/L of SAC (Text S2) before the transformation experiment. However, pre-exposure of plasmid had no significant effect on the transformation frequency, compared to the control (*p* = 0.394, Fig. S9), indicating that artificial sweeteners had no substantial damage on the plasmid.

The successful uptake of extracellular pWH1266 plasmid by *A. baylyi* ADP1 was confirmed by multiple approaches. First, DNA extraction from colonies growing on selective plates revealed the presence of plasmid DNA in the recipient cells but no plasmid DNA was detected in control cells (Fig. S10a). Second, PCR assays for ARGs *bla*_*TEM-1*_ and *tetA*, which are present on the pWH1266 plasmid, revealed that each recipient contained the two ARGs but the controls did not (Fig. S10b). Finally, the IC90 was calculated for each recipient and revealed that the transformants (the recipient with pWH1266 plasmid) had a higher resistance to antibiotics such as ampicillin (Amp, > 140 mg/L) and tetracycline (Tet, 7.5 mg/L), compared to the parent *A. baylyi* ADP1 lacking pWH1266 plasmid (10 mg/L of Amp; 1.5 mg/L of Tet) (Fig. S10c).

To further validate the phenomenon incurred by these artificial sweeteners, we also tested with a Gram-positive human pathogen *Bacillus subtilis* as the recipient. We found that in this recipient, the tested sweeteners at 30 mg/L or higher can significantly promote the horizontal transfer of extracellular ARGs into this recipient via transformation. For example, up to 3.0-fold increase was induced within *Bacillus subtilis* by SUC at 60 mg/L (*p* = 0.003; Fig. 1b). Moreover, we established an in vitro model by using mice faecal microbiome as the recipient to investigate if these artificial sweeteners could promote transformation at a community level. The successful transfer of *gfp*-encoded pKJK5 plasmid in faecal bacteria was confirmed (Fig. S10d). Around 5.1-fold increase was observed within faecal bacteria by SUC at 300 mg/L (*p* = 0.000012; Fig. 1c). By contrast, neither sucrose nor glucose could promote transformation frequency within faecal bacteria community (*p* > 0.335; Fig. S11). Taken together, these results suggest that the artificial sweeteners (SAC, SUC, ASP and ACE-K) indeed enhance the horizontal transfer of extracellular ARGs among competent cells with higher potentials than sugars, in both Gram-negative and positive strain levels, as well as at a community level.

### Artificial sweeteners affect cell envelope permeability

**Fig. 2:**
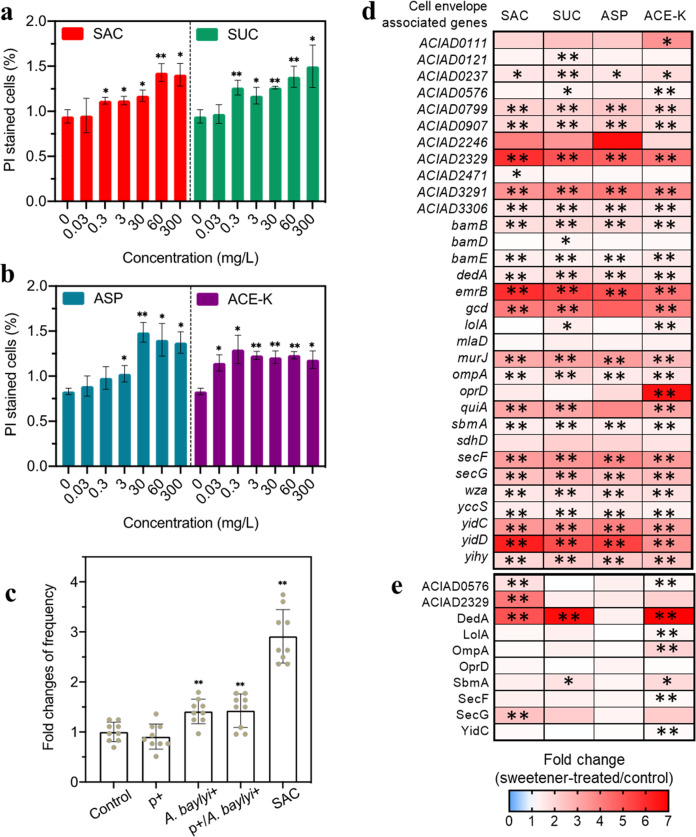
Artificial sweeteners increased the cell envelope permeability of *A. baylyi* ADP1. **a** Fold changes of cell envelope permeability induced by SAC and SUC (*N* = 3). **b** Fold changes of cell envelope permeability induced by ASP and ACE-K (*N* = 3). Approximately 3000 events in total were detected and were analyzed for each sample from **a** and **b**. **c** Influence of various pre-exposure of *A. baylyi* with 3 mg/L SAC on the fold changes of transformation frequency (*N* = 9). p+, means that the plasmid (p) pWH1266 was pre-exposed with 3 mg/L SAC for 2 h before transformation experiment without SAC or chlorine; *A. b*+, means that the recipient *A. baylyi* was pre-exposed with 3 mg/L of SAC for 2 h but was not exposed to SAC during transformation; SAC, means that no pre-exposure was conducted and the mixture of plasmid and the recipient was directly treated with 3 mg/L SAC for 6 h transformation. **d** Fold changes in relative expression levels of cell envelope-associated mRNA genes in *A. baylyi* after exposed to artificial sweeteners 2 h (*N* = 3). **e** Fold changes in cell envelope-associated proteins in *A. baylyi* after exposed to artificial sweeteners 6 h (*N* = 3). Significant differences between sweetener-treated groups and the control group were expressed with *p_*-adj*._ < 0.05 and **p_*-adj*_ < 0.01.

As an essential organelle for the Gram-negative bacteria, the cell envelope serves as a robust barrier against the transport of extracellular DNA into the inner cell [35, 36]. We hypothesized that the increase in transformation observed in the presence of the artificial sweeteners was due in part to an increase in cell envelope permeability. To test this hypothesis, we exposed *A. baylyi* ADP1 to four artificial sweeteners at the concentrations indicated previously and assessed cell envelope permeability using propidium iodide (PI) staining. Flow cytometry analyses revealed that after 2 h exposure, the PI stained bacterial increased in a concentration-dependent manner, with a 1.2- to 1.5-fold change under exposure to 300 mg/L of all four artificial sweeteners (Fig. 2a, b). Compared to artificial sweeteners, sucrose and glucose did not significantly change cell envelope permeability (*p* > 0.383; Fig. S12).

To solidate the effect of such increased cell envelope permeability on the transformation frequency, we explored the effect of pre-exposure of *A. baylyi* ADP1 with 3 mg/L SAC, at which concentration SAC was found to significantly increase cell envelope permeability (Fig. 2a; the pre-exposure details shown in Text S2). There was a significant increase (by 1.5-fold compared to the control, *p* = 0.004; Fig. 2c) of transformation frequency when only *A. baylyi* ADP1 was pre-exposed with SAC for 2 h. This suggests that the pre-exposed bacteria were more capable to acquire free DNA molecules, compared to the control.

To further investigate the molecular bases for the increase in transformation frequency after exposure to artificial sweeteners, we examined gene expression and the protein content of cells before and after exposure to 30 mg/L of all artificial sweeteners. Transcriptomic analyses of total cellular mRNA revealed that expression of cell envelope related genes was significantly regulated after exposed to all artificial sweeteners (Fig. 2d; Supplementary Data File 1). For instance, *ompA*, encoding an integral outer membrane protein, and the *ACIAD2329* gene encoding a putative membrane transporter were significantly upregulated (by 1.5- to 1.9- and 4.2- to 5.9-fold, respectively) in the cells treated by all artificial sweeteners. Expression level of the gene *oprD* that encodes outer membrane porin was also significantly increased (up to 6.6-fold when treated with ACE-K). Correspondingly, proteomic analysis revealed that cell envelope associated proteins, including integral outer and inner membrane proteins, were produced at higher levels after cells were exposed to artificial sweeteners (Fig. 2e; Supplementary Data File 2). For example, levels of the putative membrane transporter ACIAD2329 were increased 4.2-, 1.5-, 1.5-, and 2.1-fold compared to the control after exposure to SAC, SUC, ASP, and ACE-K, respectively. These genes upregulation was consistent with the observation of enhanced cell envelope permeability, showing that the upregulation of cell membrane related mRNA genes and proteins induced by artificial sweeteners contributed to the increased cell mem envelope permeability. In addition, the gene encoding lipid A hydroxylase LpxO (remodelling or modification of LPS) was significantly upregulated (*p* = 1.4 × 10^−4^ ~ 1.5 × 10^−4^), while the gene encoding lysophospholipid acyltransferase (responsible for phospholipid biosynthesis and membrane asymmetry and diversity) was downregulated (*p* = 1.4 × 10^−4^ ~ 1.5 × 10^−4^) (Supplementary Data File 1).

Moreover, the presence of these sweeteners also induced the upregulation of genes and proteins responsible for efflux pump (Fig. S13; Supplementary Data File 1 and 2). For example, the expression of multidrug efflux pump gene *emrB* was significantly (*p* = 1.4 × 10^−4^ ~ 1.5 × 10^−4^) upregulated by 5.9-, 5.5-, 5.1-, and 4.2-fold after exposed to SAC, SUC, ASP, and ACE-K, respectively. Collectively, artificial sweeteners can alter cell envelope permeability and especially can exert an antibiotic-like activity.

### Artificial sweeteners influence competence

Previously, it was documented that *A. baylyi* ADP1 requires the T4P DNA uptake apparatus and Com machinery to enable natural transformation [9, 37, 38]. Given that the artificial sweeteners promoted natural transformation, we hypothesized that the genes encoding the T4P and Com systems would be upregulated.

**Fig. 3:**
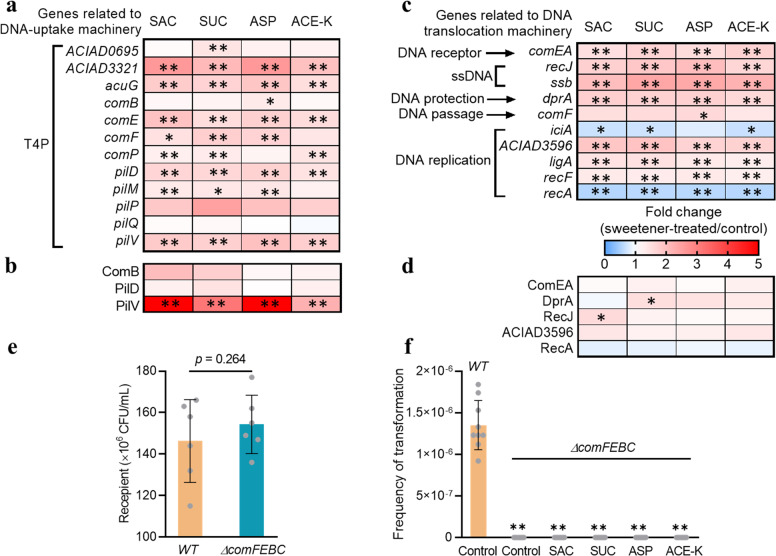
Transcriptional and proteomic analyses for *A. baylyi* ADP1 under exposure to artificial sweeteners (*N* = 3). **a** Fold changes in expression of mRNA genes related to DNA uptake machinery (T4P). **b** Fold changes in expression of DNA uptake-related proteins. **c** Fold changes in expression of mRNA genes related to DNA translocation machinery. **d** Fold changes in expression of proteins related to DNA translocation machinery. **e** The colony number of two recipients (*A. baylyi*, *WT*; the mutant, *∆comFEBC*) after 6 h transformation (*N* = 6). **f** The spontaneous transformation frequency when *WT* and *∆comFEBC* were used as recipients (*N* = 9), in the absence or presence of artificial sweeteners (30 mg/L). Significant differences were expressed with *p_*-adj*._ < 0.05 and **p_*-adj*_ < 0.01.

Our transcriptomic data revealed that the T4P-related genes were upregulated (Fig. 3a; Supplementary Data File 1). For example, the expression of *pilV* was significantly increased by 1.8-, 1.7-, 2.0-, and 1.8-fold after treatment with 30 mg/L of SAC, SUC, ASP, and ACE-K, respectively. The expression of *pilD* was also upregulated (up to 1.7-fold increase for four sweeteners). Genes encoding prepilin-type N-terminal domains also showed upregulation. The expression of *acuG* that encodes a thin pilus adhesin AcuG to stabilize the assembled fibres across the membrane during T4P process was significantly increased, with 1.8-, 1.7-, 1.9-, and 1.5-fold induced by those artificial sweeteners. Correspondingly, the upregulation of DNA-uptake related proteins was also found (Fig. 3b). For example, expression of type 4 fimbrial biogenesis protein PilV was significantly increased with 3.2- (SUC) and 2.2-fold (ACE-K), respectively (Supplementary Data File 2).

Internalization or translocation of DNA to the cytoplasm involves the DNA binding and DNA replication. In this study, genes encoding those components were also significantly regulated (Fig. 3c; Supplementary Data File 1). For example, expression of *comEA* was significantly increased (1.5- to 1.7-fold upregulation, *p* = 0.000141–0.00353) by all four sweeteners. The expression levels of genes related to single strand DNA (ssDNA) (encoded by *recJ* and *ssb*), DNA-protecting protein DprA (encoded by *dprA*), and energizing the incoming DNA through DNA channel (by *comF*) were also upregulated in the sweetener-treated groups. The other strand of the DNA molecule could be degraded in periplasm and be possibly secreted by pseudopilus. The expressions of the key DNA replication genes (*ACIAD3596*, *ligA* and *recF*) were upregulated. In contrast, genes responsible for the inhibitor of chromosomal replication (*iciA*) and for chromosomal recombination (*recA*) were significantly downregulated (Fig. 3c). Consistently, expression of related proteins such as ComEA and RecJ was upregulated, while the protein RecA was downregulated (Fig. 3d; Supplementary Data File 2).

To further confirm the critical roles of *com* operons in DNA binding and transport during transformation, we used a mutant *∆comFEBC* for the same transformation assay. Results show that a comparable number of *A. baylyi* ADP1 (WT) and its mutant (*∆comFEBC*) were observed after a 6 h transformation assay (Fig. 3e). However, no *∆comFEBC* colonies were recovered on selective plates, indicating that the transformation frequency was much lower than that wild-type recipient (Fig. 3f). Same results were also obtained when exposing the mutant to the artificial sweeteners during transformation experiments. These results suggest that these *com* operons are indispensable for transformation [25]. Collectively, these results showed that artificial sweeteners activate the T4P and Com machinery in *A. baylyi* ADP1, enabling uptake the extracellular pWH1266 plasmid.

### Artificial sweeteners affect plasmid persistence in transformed cells

**Fig. 4:**
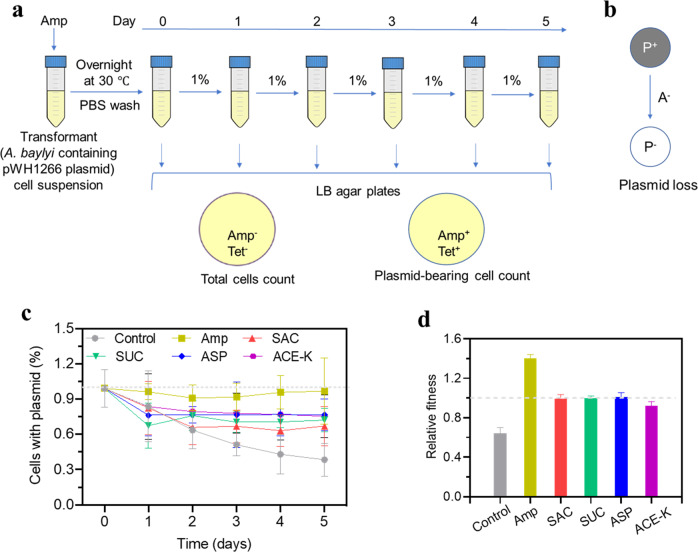
Plasmid persistence in bacterial cells. **a** Set-up of plasmid persistence assays with artificial sweeteners treatments. Every round, 1% of stationary-phase cultures were grown in fresh medium containing artificial sweeteners 24 h and were then spread on LB agar plates. Total cell number was obtained from the plate that contained neither Amp nor Tet, while plasmid-bearing cell count was conducted from the plate containing Amp. **b** Shift of bacteria cell between plasmid-bearing cells (P^+^) and plasmid-free cells (P^-^) in the absence of antibiotic (A^-^). **c** Time course analysis of plasmid persistence assay in LB media containing 30 mg/L of each artificial sweetener (*N* = 6). A negative control was set up in the absence of artificial sweeteners nor antibiotics. A positive control was also set up by adding 0.5 mg/L of Amp to pose a selective pressure on cell growth but with little killing effect on the plasmid-free strain. At each time point, colonies were enumerated from LB agar plates with or without Amp to analyse the percentage of plasmid-maintaining cells. Initial culture was plasmid-maintaining transformant cell. Error bar means the standard deviation. **d** Relative fitness (mean ± standard error of the mean) of the 5-day evolving transformants under exposure to artificial sweeteners.

The development of antibiotic resistance normally confers a fitness cost to the bacteria [39, 40]. Specifically, the carriage of mobile genetic elements (e.g., plasmid), which often contain multiple ARGs, can make microbes less fit than their susceptible counterparts. Bacteria may regain fitness by losing the genetic elements when the antimicrobial stress is removed. To understand the persistence of the acquired non-conjugative pWH1266 in transformant cells and the effect of those artificial sweeteners on the plasmid maintenance, we performed a plasmid persistence assays for transformant cells in LB media over 5 days (Fig. 4a). At the end of each day, we quantified the total cells (either plasmid-bearing and plasmid-free cells) and the fraction of plasmid-bearing cells by plate culture. We found that in the presence of Amp, the fraction of plasmid-bearing cells levelled off (~100%) throughout the assays. However, in the absence of antibiotic (Amp), pWH1266 plasmid could be lost (Fig. 4b), as the fraction of plasmid-bearing cells was significantly decreased to below 40% from Day 0 to Day 5 (the control curve in Fig. 4c). This indicates that for a non-conjugative plasmid, the absence of antibiotics could lead to resistance reversal. In contrast, a high fraction (>70%) of cells still maintained the plasmid in the assays of artificial sweeteners exposure, even if it comes at a fitness cost to the host. These results suggest that artificial sweeteners, like antibiotics, play a role in plasmid persistence.

In addition, we estimated the relative fitness of bacteria that had acquired the plasmid under exposure to these artificial sweeteners during a course of 5 days. Bacteria that had acquired the pWH1266 plasmid demonstrated a relative low fitness (0.64, Fig. 4d) compared to the host. In contrast, increases in relative fitness were observed in sweetener-treated groups (~1.00), which was also found in Amp-treated group. This suggests a higher fitness for transformants under frequent exposure to artificial sweeteners.

### Artificial sweeteners induce cumulative effects on natural transformation

**Fig. 5:**
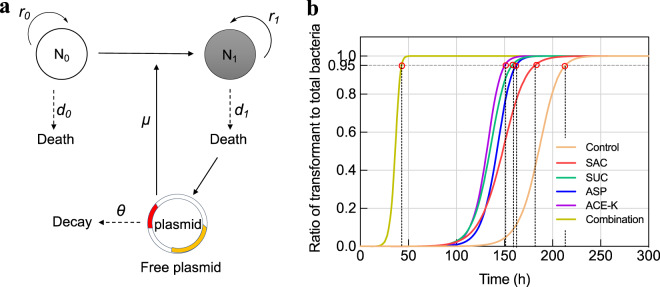
The simulated cumulative effects of artificial sweeteners (30 mg/L) on the transformation frequency (indexed as fraction of transformant to total bacteria) at different lapsed time periods. **a** Schematic diagram of modelling plasmid dynamics in a single species. The recipient (the wild-type strain, *N*_0_) uptakes free plasmid through transformation at a rate constant *μ* to become transformant (*N*_1_). The recipient and transformant grow at their own rate constant *r*_0_ and *r*_*1*_, respectively. Both strains turnover at rate constant *d*_0_ and *d*_*1*_. The acquired plasmid could be released from dead transformant cell and can be transformed to the recipient. The plasmid could be decayed at a rate constant *θ*. **b** Simulated curves of transformant number (indicated as fraction of transformant to total bacteria). The control group means the spontaneous transformation frequency without any sweetener exposure. Pink circles indicate the stability time of transformation in each group. Parameter values used for modelling are shown in Table 1.

In the laboratory assays, the time for the transformation normally lasts serval hours [41, 42], or up to one day [43]. In the present study, the duration of transformation was 6 h, at which artificial sweeteners were found to promote the occurrence of transformation event. However, little is known about how the transformation process proceeds during a long-term period. Given that artificial sweeteners frequently co-exist and are less prone to be degraded in the human gut tract and other environments (e.g., WWTPs), we developed a mathematical model (Fig. 5a; Text S9) to predict the long-term impacts of four artificial sweeteners (30 mg/L) and their combinations on the transformation frequency. The estimated number of the wild-type bacterium rapidly decreases and finally all become transformants (Fig. S14). Meanwhile, free plasmid can be released from the transformants (plasmid loss or cell lysis), and the number of free plasmid copies keeps increasing together with the transformant number.

We also found that exposure to artificial sweeteners affected the time for reaching the final stable value (i.e., the stability time). Compared to that in the control group (215 h), the stability time in sweetener-treated groups was greatly shortened to 152 h (Fig. 5b; Table S11). This process can be further accelerated by the combination of four sweeteners, with only 44 h required for reaching the stable value. It is worth mentioning that the stability time is determined with both the transformation frequency and death rate in the model. Two variables (transformation frequency *K*_μ_* and death rate Kd*) involved in the simulation were quantitively analyzed. The predicted transformation frequency (*K*_μ_*) in the long run showed an increase under exposure to artificial sweeteners, compared to the control group (Table S11). For example, SAC, SUC, ASP and ACE-K would induce 2.3-, 1.9-, 1.5- and 1.6-fold increase in transformation frequency, respectively. The increase would reach ~11.0-fold when the combined effect of four sweeteners was considered. Together, artificial sweeteners are predicted to continually enhance transformation process in aspects of both frequency and transfer rate, and the combination of four sweeteners would further accelerate transformation dynamics under long-term exposure conditions.

## Discussion

As one of HGT pathways, transformation enables the competent bacterial species to evolve as resistant bacteria, regardless of phylogenetic relationship. Although natural competence for transformation has been the focus for almost 100 years, this process is still not fully understood. In addition, it is well acknowledged that the HGT process is commonly promoted by antibiotics. However, little is known whether the widely used artificial sweeteners could speed up natural transformation. In the present study, we found that all artificial sweeteners significantly promote transformation frequency of extracellular DNA in both Gram-positive and Gram-negative bacteria, and this phenomenon also occurs at a community level in terms of an in vitro model. Fold change of transformation frequency induced by artificial sweeteners is up to 5.1-fold increase (by SUC), which is comparable to about 2.0- and 6.0-fold increase induced by antibiotics vancomycin and fosfomycin [44]. It should be noted that such increase is not associated with available carbon sources provided by artificial sweeteners, because they are not metabolically used by the tested bacteria (Text S11; Fig. S15b). In contrast, the commonly-used sugars (sucrose and glucose) can slightly enhance transformation among *A. baylyi* ADP1, which is due to the enhanced bacterial competence by nutrients [45, 46]. Indeed, the concentrations of sugars significantly decline (Fig. S15a) after transformation assay, indicating that sugars are metabolized by bacteria as nutrients or carbon sources.

### Mechanisms for enhanced transformation by artificial sweeteners

**Fig. 6:**
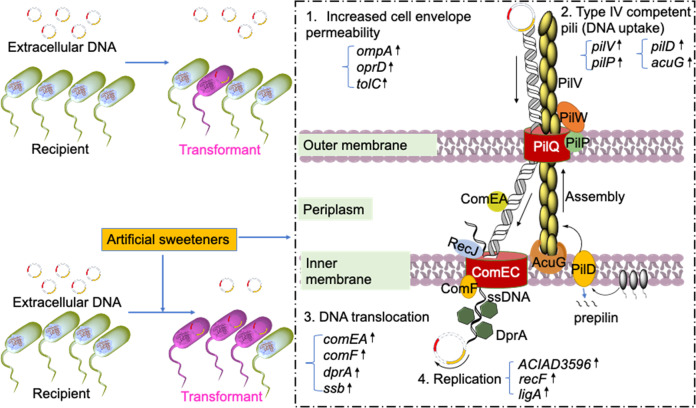
A proposed model for transformation process in competent bacterium *A. baylyi* ADP1. Transformation of extracellular DNA in bacterial cells is promoted by artificial sweeteners, which activate competence system (e.g., type IV competent pili formation, DNA translocation and replication) in bacteria. First, artificial sweeteners increase cell membrane permeability. Genes (e.g., *ompA*, *oprD*, and *tolC*) encoding outer membrane proteins were significantly up-regulated. The increased permeability would make free plasmids easier to be taken up by *A. baylyi*. Second, the competence ((e.g. type IV competent pili formation and DNA translocation) of *A. baylyi* was enhanced by artificial sweeteners, as confirmed by the up-regulation of related mRNA genes and proteins. Type IV competent pili-related genes (*PilV* encoding pilus fibre, *pilD* encoding prepilin peptidase, *PilP* encoding pilot protein, and *AcuG* encoding pilus adhesin protein) were increasingly expressed to promote free plasmid uptake. Afterwards, the plasmid was more efficient to be internalized by DNA translocation machinery, of which DNA receptor *ComEA* and DNA transport channel (*ComF*, *DprA* and *Ssb*) were up-regulated. The arrived DNA replicates (DNA replication related genes *ACIAD3596*, *RecF*, and *LigA* were up-regulated) into an independent circular DNA molecule (plasmid).

The underlying mechanisms of artificial sweeteners in the enhancement of natural transformation were explored by multiple analyses such as cell membrane permeability, whole-genome RNA sequencing analysis of gene expression and correspondingly quantitative proteomic response analysis. Our data suggest the mechanisms including increased cell envelope permeability, the activated T4P and Com machinery, together underlying the artificial sweeteners-promoted transformation in natural competent bacteria (Fig. 6).

First, we found that artificial sweeteners increased cell envelope permeability, which will facilitate natural competent bacterial cells to uptake free plasmids. As an entry of extracellular substance, bacterial cell membrane plays a critical role in the acquisition of external free plasmid [47, 48]. In the present study, cell envelope permeability was significantly increased under exposure to four artificial sweeteners. This increase can be further confirmed by the results of the whole genome gene and protein sequencing analyses related to bacterial cell envelope. We also found that artificial sweeteners damage cytoplasmic membrane via remodelling LPS/lipid A in membrane, further supporting the increased cell envelope permeability by the tested sweeteners. It is suggested that artificial sweeteners increase bacterial cell envelope permeability, thus enabling *A. baylyi* ADP1 to uptake free extracellular DNA more easily. This correlation can be supported by the higher transformation frequency induced by SUC than that induced by both sucrose and glucose, which did not increase cell membrane permeability. Previous studies have reported that antibiotics can damage cell envelope or block cell membrane synthesis to increase cell envelope permeability and then promote gene transfer by transformation [49, 50]. Recent studies also ascribed the increased transformation frequency to the increased cell envelope permeability by disinfectant chlorine [41, 51].

In natural transformation, the activation of SOS response and competence can be pathways that enable the host to acquire extracellular DNA for repairing or development of antibiotic resistance [52]. However, *A. baylyi* ADP1 does not have lexA homologues, and therefore no corresponding SOS response [53, 54]. In our study, we did not detect any reactive oxygen species (ROS, triggering SOS response) overproduction after exposure of bacteria to artificial sweeteners (Text S10; Supplementary Data File 1; Figs. S16 and S17). The downregulation of genes *recA* and *dinB* (encoding DNA damage-inducible protein) expression (Fig. 3c and Supplementary Data File 1) in response to artificial sweeteners also supported that SOS response may not be involved in the transformation process. Instead, the competence has been reported to be an SOS response substitute in response to stress in transformable species [8, 18, 55, 56] and the competence-related genes and proteins such as *com* operons that encode DNA uptake apparatus (T4P) and DNA translocation machinery were increasingly expressed after exposing *A. baylyi* ADP1 to artificial sweeteners (Fig. 3a-d). Thus, our findings suggest that the competence, rather than SOS response contributes to the ARG transfer via transformation associated with artificial sweeteners. This is also in agreement with a previous research, in which *A. baylyi* did not possess an SOS response to water disinfection by-products that increased natural transformation [57].

Moreover, T4P is normally functioned on the surface of Gram-negative or positive species and is initialized with the assembly of type IV pilin in the form of prepilins. The prepilins interact with the cytoplasmic membrane via their hydrophobic domains (amino- and carboxy-terminal domains) and can be then processed by prepilin peptidase that resides in the cytoplasmic membrane [58, 59]. The prepilin peptidase promotes the translocation of mature pilins across the outer membrane and the assembly of pilins into pilus filament protruded from the membrane. The filament interacts with outer membrane via its N-terminal domains and finally could become pilus tip. In this study, both transcriptional and proteomic analyses have showed that T4P-related genes and proteins were up-regulated significantly.

### Artificial sweeteners can play antibiotic-like roles

For the natural transformation, antibiotic stress can induce transformability in bacterial cells [8]. Unlike their parental strains, transformants can adapt to the stress and can be a later dominant population in the community. Under antibiotic stress, transformants will maintain their antibiotic resistance because of its fitness to survival. Thus, the functional roles of antibiotics can be reflected by the enhancement of transformation frequency, population dynamics and plasmid persistence. In this study, we found that artificial sweeteners enhanced transformability in *A. baylyi* cells after 6-h treatment. This can also be verified with the modelling results that predicted cumulative effects on natural transformation under long-term exposure conditions (over 300 h) (Fig. 5). As well, transformants can become the dominant species in a long-term run. We also found that all artificial sweeteners activated efflux pump, which is normally adopted by bacterial cells to reduce intracellular antibiotic levels [60].

Furthermore, artificial sweeteners played an antibiotic-like role in plasmid persistence. It is estimated that almost half of all plasmids are non-transmissible [61]. Co-evolution between the host bacteria and plasmid modulates plasmid persistence that matters the dissemination of antibiotic resistance. We found that the acquired pWH1266 plasmid from the spontaneous group without antibiotic nor artificial sweeteners can be lost (Fig. 4c). In the presence of artificial sweetener-mediated pressure, the acquired plasmid can be well maintained. This is similar to the group treated with ampicillin, by which the positive selection offsets plasmid loss during segregation [62]. In addition, we found that exposure to artificial sweeteners increases relative fitness of the acquired plasmid to the host (Fig. 4d), which is consistent with the results of antibiotic (Amp) treatment. In addition to fitness cost on the host, the long-term maintenance of plasmid also requires the plasmid to own active systems such as plasmid partitioning systems (ParA/ParB) that play critical roles in stable maintenance of plasmids [63]. However, this active partitioning is normally carried by transmissible plasmids (i.e., RK2 and pKJK5) from IncP-1 incompatibility group. As a non-transmissible plasmid, pWH1266 has no *par* operons indicated by RNA sequencing analysis in our study. Consequently, the pWH1266 plasmid cannot be tethered during cell division and daughter cells may not receive a copy of plasmid. Together with the long-term effects on transformation dynamics suggests that artificial sweeteners can play an antibiotic-like role in promoting plasmid persistence in the host and would maximize the spread of ARGs in the environment.

### Implications to public health and environmental risk management

Considering that artificial sweeteners are commonly applied in food and beverages and that the consumption of those sweeteners is soaring, the effects of artificial sweeteners on the spread of antibiotic resistance should not be ignored. As pools of various microbiota and ARGs, the human gut system will definitely be one of hotspots for exchanging ARGs through natural transformation. It is estimated that our human gut system consists of 10^14^ bacteria involved in multiple health-related interactions [64, 65]. This microbiota system is a huge pool of bio-genome including ARGs and can serve as a transporter for DNA exchange [66]. Previous studies have confirmed the occurrence of HGT in the gastrointestinal track [33, 67–69]. Generally, the gut bacteria colonize tract surface and are sensitive to the conditions. A previous study reported that artificial sweeteners induced antibiotic-like alteration of gut microbiota [12]. This causes a concern of potential adverse effects brought by the popular consumption of artificial sweeteners. Our present study demonstrates that artificial sweeteners can promote horizontal transfer of ARGs among competent *Bacillus subtilis* and gut microbiome. To this end, re-evaluation of the potential antibiotic-like side effects of artificial sweeteners becomes a necessity regarding the public health associated with their consumption. Further in vivo transformation experiments should be conducted to validate potential risks of artificial sweeteners to human or animal gut health. Clinically-relevant plasmids that carry resistance to other antibiotics (e.g., colistin as an antibiotic of last resort for human health) could also be adopted to further generalize the role of artificial sweeteners in the spread of various ARGs among gut microbiome. In addition, considering that these sweeteners are normally excreted in urine, it will be also necessary to evaluate if they could shift resistance profiles in the human urinary tract infections.

Moreover, artificial sweeteners might have played a role in the dissemination of antibiotic resistance in the environment (e.g. WWTPs), which should not be overlooked, neither. In this study, the dose of artificial sweeteners (0.3–3 mg/L) is environment-related range (2.5 mg/L in WWTPs [70]). The concentrations of artificial sweeteners in the environment would be higher because of persistence, accumulation and the growing consumption of artificial sweeteners [71–73]. Moreover, extracellular DNA molecules are very abundant (over 100 µg/g dry sludge [74] or up to 30 ng/µL [75], which is far higher than 0.8 ng/µL used in the present study) and can be persistent over 20 weeks [76] in the environment. Our findings reveal that the long-term exposure to artificial sweeteners not only increases transformation frequency of ARGs but also stabilizes the maintenance of the acquired plasmid. Consequently, genetic transformability would be more significantly induced by artificial sweeteners, ultimately resulting in the dissemination of ARGs in the environment. This will urge to assess the possible ecological consequences associated with the discharge of artificial sweeteners into the environment.

## Supplementary information

Supplementary Information

Supplementary Data File 1

Supplementary Data File 2

## Data Availability

All RNA sequencing data have been deposited to the Gene Expression Omnibus of National Centre for Biotechnology Information (NCBI) and are accessible through the GEO series (GSE141954). The mass spectrometry proteomic analysis data have been deposited to the ProteomeXchange Consortium via the PRIDE [77] partner repository with the dataset identifier PXD027117.
