# Supplementary material for: Artificial sweeteners stimulate horizontal transfer of extracellular antibiotic resistance genes through natural transformation
Source: ISME J. 2021 Sep 1;16(2):543–54. doi: 10.1038/s41396-021-01095-6 (PMC8776823; doi:10.1038/s41396-021-01095-6)
Supplement: Supplementary file 1 — Supplementary Information [file 41396_2021_1095_MOESM1_ESM.docx]

**Text S1. Optimization of transformation time**

Several transformation experiments were conducted to optimize the transformation time. Samples (cell suspension) were collected from the transformation system (without artificial sweeteners) at different time intervals (0 h, 2.5 h, 4 h, 6 h, 8 h, 12 h, 24 h) and were spread onto the LB agar plates to count for the number of the recipient *Acinetobacter baylyi* ADP1 and the transformant. The plate for the recipient contained no antibiotics, while for the transformant contained 100 mg/L Amp and 20 mg/L Tet. After incubation at 30 ºC for a certain time, the frequency of transformation was calculated by Equation 1.

$$Frequency of transformation = \frac{[Number of transformant]}{[Number of the recipient]} (1)$$

Afterwards, the transformation rate was calculated (Equation 2) based on the above transformation frequency and the corresponding elapsed time.

$$Transformation rate = \frac{[Number of transformant]}{[Number of the recipient]\times time} (2)$$

**Text S2. Pre-exposure of plasmid and the recipient to artificial sweeteners**

To investigate whether different pre-exposure toward plasmid and the recipient affects the transformation frequency three means of pre-exposure were conducted in this study: (1) only the plasmid pWH1266 exposure to artificial sweeteners before transformation experiment; (2) only the recipient exposure to artificial sweeteners before transformation experiment; and (3) both the plasmid and the recipient exposure to artificial sweeteners before transformation experiment. According to the result in this study, we found that all types of artificial sweeteners significantly increased cell membrane permeability of the recipient (Fig. 2a, b). Herein, SAC was used as a representative of artificial sweeteners to significantly increase the permeability of the strain.

First, culture of the recipient was overnight (about 14 h) incubated at 30 ºC and was then incubated (1% incubation) in LB media for further growing. Afterwards, the cell pellets were collected by centrifuge at 5000 rpm for 5 min and were washed by PBS two times. The cells were vortexed to resuspend in the PBS solution containing 40 mg/L COD and were then adjusted to reach an OD600 value of 1.10. Prior to transformation experiment, the plasmid pWH1266 was treated by 3 mg/L SAC for 2 h (pre-exposure 1). For pre-exposure 2, the recipient *A. baylyi* was treated with 3 mg/L SAC for 2 h and then was centrifuged and washed to remove any SAC residue, and finally were resuspended in PBS solution containing plasmid. While for pre-exposure 3, the mixture of plasmid and recipient was directly exposed to SAC for 6 h transformation. Meanwhile, a control (H_2_O addition) was set up. After 6 h transformation, the numbers of the recipient and the transformants were counted by spreading a certain volume of mating systems to the corresponding selective LB agar plates that contained different types of antibiotics.

**Text S3. *In vitro* transformation in faecal bacteria**

Faeces were collected from 6~8-week-old C57BL/6 oligo microbiota mice housed with food and water in the animal facility of the Institute for Molecular Bioscience. The collected faeces were immersed in PBS (0.1 g/mL) and were homogenized by a vortex (maximal speed, 5 min) [1]. The homogenates were centrifuged at 500 × g for 30 s to remove larger debris [2]. The IncP-1ε broad host range plasmid pKJK5 that is tagged with a *gfpmut3b* gene encoding green fluorescent protein (GFP) and carries an antibiotic resistance gene (Kan^R^) was used as extracellular DNA. Once successful transfer of plasmid pKJK5 to a faecal bacterium, *gfp* can be expressed and can produce green-fluorescent cells. Based on such fluorescent reporter, faecal bacteria that successfully receive plasmid pKJK5 (becoming transconjugants) can be sorted by a fluorescence-activated cell sorting (FACS) technique. The initial concentration of plasmid was 1.0 ng/*μ*L. Transformation events were quantified by a CytoFLEX S flow cytometer (Beckman Colter, USA) with excitation at 488 nm and emission at 525 nm [3]. Approximately total 300,000 events were analyzed with the detection rate of around 3000 event/second.

Detection results were analyzed by FlowJo 7.6. Transformants were sorted by setting up triple gates: the gate I of forward scatter-H vs side scatter-H plot was initially set up to focus the particles with bacteria size; the gate II of forward scatter-H vs forward scatter W plot was used to target on singlet; the third gate III of 561_TexaRed-A vs 488_SYBR-A plot was used to exclude any auto-fluorescent particles from faecal samples and at the same time sort only out transformants (Figure S2). All FACS plots were conducted in bi-exponential axis in order for the full analysis. Method of triple-gated sorting was confirmed by both negative (nonfluorescent *E. coli* K-12 MG1655 wild type and faecal bacteria) and positive (transconjugant *E. coli* K-12 MG1655 with pKJK5 plasmid, generated by mating the nonfluorescent *E. coli* K-12 MG1655 wild type with red-fluorescent *E. coli* K-12 MG1655 harbouring *gfp*-pKJK5 plasmid) control samples. The recipient number was also quantified as the event number detected on the down left quadrant. The transformation frequency was calculated as the transformant number divided by the recipient number. Fold changes in transformation transfer were also calculated by normalization of the transfer ratio to the control group (without sweetener treatment) and were displayed in this study. In addition, the successful transfer of the plasmid in faecal bacteria was also confirmed by PCR assay of *gfp* in faecal bacteria before and after experiment. After transformation assay, cell pellets were collected by centrifugation (5000 × g for 5 min) and were washed three times before suspended in PBS. The collected cells were used for DNA extraction by the PowerWater® DNA isolation kit (MoBio), according to the manufacturer’s protocols. The extracted DNA samples from faecal bacteria before and after transformation assay were used for PCR assays. The gene *gfp* primer was shown in Table S2. The procedure of PCR assay was programmed as: 94 ºC for 10 min, followed by 30 cycles at 94 ºC for 1 min, at 51 ºC for 1 min, at 72 ºC for 1 min, and a final extension at 72 ºC for 7 min.

**Text S4. Measurement of cell envelope permeability**

First, artificial sweeteners or sugars (sucrose and glucose) were added to the cell suspension suspensions (approximately 10^6^ CFU/mL in PBS solution). After 2-h treatment at room temperature, the fluorescent reporter dye, propidium iodide (PI, Life Technologies, USA), was added to the solution. The final concentration of PI was 20 *μ*M. Then, the stained solution was incubated in the dark for 15 min before scanned by the flow cytometer. The fluorescence (488 nm/561 nm) intensity or the percent of stained cell was measured as the indicator of cell envelope permeability. The intensity was deducted by that of unstained cell suspension. Meanwhile, the negative (Milli-Q water instead of sweeteners) and positive (cell suspensions were heated at 80 ºC for 2 h) controls were prepared. The flow rate was set as 10 *μ*L/min, and the event (or cell) detection rate was approximately 200 events/second. Approximately 3000 events in total were detected and analyzed for each sample. All samples were prepared in biological triplicates.

**Text S5. Analysis of growth curves**

To investigate the effects of artificial sweeteners on bacterial growth, the growth curves were plotted as the OD_600_ value dynamically recorded every 5 min for 16 h of incubation at 30 ºC with shaking in a CLARIOstar Multimode plate reader (BMG LABTECH). For each treatment, the overnight culture was initially diluted 1000 times with fresh LB medium. A volume of 3 mL diluted cell suspension (~10^5^ CFU/mL) was used for inoculation, in triplicate, of 135 *µ*L of LB medium and 15 *µ*L of solutions that contained different concentrations of artificial sweeteners (60 and 300 mg/L) using 96-well flat-bottom plates. The control was also set up with the addition of 15 *µ*L of sterilized deionized water.

**Text S6. Plasmid extraction and PCR assays of resistance genes**

First, five transformant colonies were randomly selected from the selective plates and were overnight incubated in LB media. Plasmids from the collected strains were extracted by GeneJET Plasmid Miniprep Kit (Life Technologies, Australia), according to the manufacturer’s instructions. After that, the presence of plasmid in transformants was initially confirmed by the agarose gel electrophoresis. Meanwhile, the extracted plasmid was also used for the detection of *bla_TEM_* and *tetA* by a qualitative PCR technique. The 20 *μ*L volume reaction assays, which consists of 1 *μ*L of DNA sample, 10 *μ*L of Platinum Green Hot Start PCR 2X Master Mix (Invitrogen by Thermo Fisher Scientific), 2 *μ*L of Platinum GC Enhancer, 6.6 *μ*L of Milli-Q water, 0.2 *μ*L of *bla_TEM_* Long FW and RV for Amp gene, and 0.2 *μ*L of *tetA* Long FW and RV for Tet gene, were carried out. The procedure was programmed as: 94 ºC for 10 min, followed by 30 cycles at 94 ºC for 1 min, at 60 ºC for 1 min, at 72 ºC for 1 min, and a final extension at 72 ºC for 7 min. The amplification process was run by Applied Biosystems.

**Text S7. Protein extraction and proteomics analysis**

Bacterial cells were collected by centrifuging at 8000 × g for 5 min and were washed by PBS twice. This is followed by resuspending cell pellets in 0.5 mL PBS solution. Afterwards, the samples were stored in freezer at -80 ºC before analysis. The prepared bacterial cells were lysed by with 5% sodium dodecyl sulfate and were thoroughly sonicated to remove DNA residues. This was followed by addition of dithiothreitol (20 mM, final concentration) in order to reduce disulfides. The samples were heated at 70 ºC for 60 min and were cooled down to room temperature. After that, the samples were alkylated with 40 mM iodoacetamide (final concentration) in the dark for 30 min. The alkylated samples were treated with a final concentration of 1.2% phosphoric acid and six volumes of S-Trap binding buffer (90% methanol, 100 mM final concentration of ammonium bicarbonate, pH 7.1).

The protein solution was then loaded on the S-Trap filter and was centrifuged at 4,000 × g until all solution passed through. The S-Trap filter was washed with 150 *μ*L of S-Trap binding buffer for three times. Samples on the S-Trap filter were digested with 1 *μ*g of sequencing-grade trypsin at 47 ºC for 1 h (avoid any bubbles on the filter). The digested peptides were then eluted with 40 *µ*L of 50 mM ammonium bicarbonate, 0.1% aqueous formic acid, 50% acetonitrile and 0.1% formic acid, respectively, and were lyophilized before resuspended in 20 *µ*L of 5% acetonitrile. Analysis was performed by a liquid chromatography-tandem mass spectrometry (LC-MS/MS) using a Dionex Ultimate 3000 RSLCnano-LC system coupled to a Q-Exactive TM H-X Hybrid Quadrupole-Orbitrap™ mass spectrometer (Thermo Scientific TM).

**Text S8. Plasmid** **persistence assay**

Colonies of transformants were randomly collected from the selective plate (the spontaneous transformation group), then were overnight incubated in LB media containing 100 mg/L Amp antibiotic. Cell pellets were washed with sterilized PBS to remove antibiotic residue and were then suspended in fresh LB media. After that, 5 *μ*L of aliquot was transferred to another LB media tubes that contained 3 mg/L of each artificial sweeteners. The initial concentration of bacteria cell was ~10^6^ CFU/mL. Cell suspensions were incubated in a shaker (100 rpm) at 30 ºC for 24 h before sampling for the enumeration of cell with or without pWH1266 plasmid.

At the end of each incubation, samples were collected from the tube, diluted and spread on LB agar plates. Two types of plates were used here. One is the plate that did not contain any antibiotics used for the enumeration of total cells (both plasmid-bearing and plasmid-free cells), another is the plate that contained 100 mg/L and 20 mg/L Tet used for plasmid-bearing populations count. Meanwhile, two controls (one is the negative control without addition of artificial sweeteners and antibiotics; the other is the group with addition of 0.5 mg/L Amp) were also set up. Phenotypical mutagenesis of plasmid-free cells (susceptible to Amp and Tet) was ruled out by incubation on the selective plates for 5 days.

The fraction of cells with or without pWH1266 plasmid was quantitively measured on Day 1 (D1), D2, D3, D4, D5, respectively. Initial fraction of bacteria cells before the assays (D0) was analysed. Two control groups (no artificial sweeteners and 0.5 mg/L Amp) were also set up. The plate that contained no antibiotics was used for the total bacteria cells, while the plate containing 100 mg/L Amp was used for the cells that maintained pWH1266 plasmid. The fraction of cells with plasmid was calculated as dividing the total cell number with the number of cells that maintained plasmid.

**Text S9. Modelling of transformation dynamics by an ordinary differential equation (ODE) model**

In this study, explicit constraints of two key variables (*μ* and *d*) were set up were described below:

$$\left\{\begin{matrix} LB_{\mu}\leq K_{\mu}\leq UB_{\mu} \\ LB_{d}\leq K_{d}\leq UB_{d} \end{matrix}\right.$$

Initially, the populations satisfying the corresponding constraints were generated by a creation function and were used for the ODE simulation model. After that, the simulated values of *N*_0_ and *N*_1_ after 6 h exposure were calculated with the ode 15s (effective stiff solver) in MATLAB 2016b. During calibration process, feasible subranges of related factors were applied to effectively find the global optimization point, in order to generate the two variables (*Kμ* and *Kd*) with smaller ranges. Meanwhile, nine benchmark points (Table S3) of scale factor were used to configurate eight partitions of individual factor (Table S4 and S5). Correspondingly, the feasible subranges of genetic algorithm optimization were dependent with the two decision variables (Table S6). Note that these subranges must include the benchmark point (*L_μ_, L_d_*) that corresponds to area *Ω_μ_-Ω_d_* during optimization.

The objective functions for calculating (*L_μ_, L_d_*) can be referred to

$$\begin{matrix} \min LS\left(R_{\mu}\cdot\mu_{ref},R_{d}\cdot d_{ref}\right)=\alpha\cdot\left[N_{0,obs}\left(6\right)-N_{0,sim}\left(6\right)\right]^{2}+\beta\cdot\left[N_{1,obs}\left(6\right)-N_{1,sim}\left(6\right)\right]^{2} \\ \left\{\begin{matrix} R_{\mu}\in\Phi_{\mu} \\ R_{d}\in\Phi_{d} \\ \Phi_{\mu}=\Phi_{d}=\left\{\begin{matrix} 0.05 & 0.1 & 0.2 & 0.5 & 1 \\ 2 & 5 & 10 & 20 & \end{matrix}\right\} \end{matrix}\right. \end{matrix}$$

The search ranges *Ω_μ_-Ω_d_* for both two factors were based on the point (*L_μ_, L_d_*) and were decided with all the combination (8×8) of scale factors. Parameters related in the simulation model were summarized in Table S7. The optimal *L_μ_* and *L_d_* values, search ranges, lower and upper bounds of both key factors under different conditions were calculated and summarized in Table S8. All settings in the off-the-shelf Optimization Toolbox 7.3 were shown in Table S9.

**Text S10. Detection of oxidative stress induced by artificial sweeteners**

To explore whether oxidative stress could be induced by artificial sweeteners and could play a significant role in promoting transformation process, intracellular reactive oxygen species (ROS) was detected by the DCFDA cellular ROS detection assay kit (Abcam, UK) and by a CytoFLEX S flow cytometer (Beckman Coulter, USA) [3]. The initial cell concentration of suspensions was adjusted by PBS containing 40 mg/L COD to about 10^6^ CFU/mL before adding the fluorescent reporter dye DCFDA (2’,7’-dichlorofluorescein diacetate). The final concentration of the reporter dye was 20 *μ*M. After that, the suspensions were incubated for 30 min staining at room temperature in the dark without shaking. This is followed by adding various concentrations (0, 0.03, 0.3, 3, 30, 60 and 300 mg/L) of artificial sweeteners. After 2 h treatment, cell suspensions were run by the cytometer with excitation at 488 nm and emission at 525 nm. The fluorescence intensity of sweeteners-treated groups was deducted by that of unstained (without fluorescent reporter dye) cell suspension and was recorded to represent the percent of ROS production level. Both negative (Milli-Q water) and positive (3% hydrogen peroxide, final concentration) controls were simultaneously prepared throughout the ROS detection. All samples were prepared in biological triplicates.

To confirm whether the intracellular Fenton reaction was triggered after ROS production, we used 2,2’-dipydyl (5 mM, final concentration, sigma) as an iron chelator in order to chelate iron ions before adding artificial sweeteners (may induce ROS production). Other procedures were the same as described above. Finally, ROS production level in each cell suspension was detected.

**Text S11**

**LC-MS analysis of artificial sweeteners**

The concentrations of artificial sweeteners before and after transformation experiment (6 h) were quantified with LC-MS analysis, according to our previous study [3]. Typically, *A. baylyi* ADP1 suspension was prepared with PBS and the initial cell density was maintained at the same level as transformation experiment. After 6 h contact, the supernatant was collected by filtration with 0.22 *µ*m membrane filters. Meanwhile, a blank with no addition of sweeteners and a positive control with only artificial sweeteners were prepared.

Artificial sweeteners from the filtrate were extracted passage through Oasis HLB solid phase extraction (SPE) cartridges. The eluent was concentrated using nitrogen stream and were analyzed by by an AB 4000 Qtrap (AB Sciex, Carlsbad, CA, USA), interfaced with an Agilent 1200 Series HPLC system (Santa Clara, CA, USA).

**Optimization of transformation time.** The time for transformation model of pWH1266 plasmid uptake by *Acinetobacter baylyi* ADP1 was optimized. We found that the frequency of transformation significantly increased (*p* = 0.000 ~ 0.010; Fig. S1a) with the increasing of transformation time. Within the first 4 h, the transformation frequency was 10^-7^ per recipient. This dramatically increased to over 10^-6^ per recipient after 6 h or longer. The fitting result also indicated that the dramatic increase of transformation frequency occurred after 4.25 h contact. Although there is an increase of transformation frequency after 24 h contacting time between *Acinetobacter baylyi* ADP1 and pWH1266 plasmid, the transformation rate (one transformant per recipient per hour) significantly declined (*p* = 0.002; Fig. S1b) in comparison with that at 12 h. Indeed, the transformation rate reached the highest (3.7×10^-7^/recipient/hour) and there is no significant change (*p* = 0.937 ~ 0.981) when the transformation time ranged from 6 to 12 h. Collectively, results indicated that the transformation process was much more active after 6 h contacting time, which was suggested for use in the next transformation model.


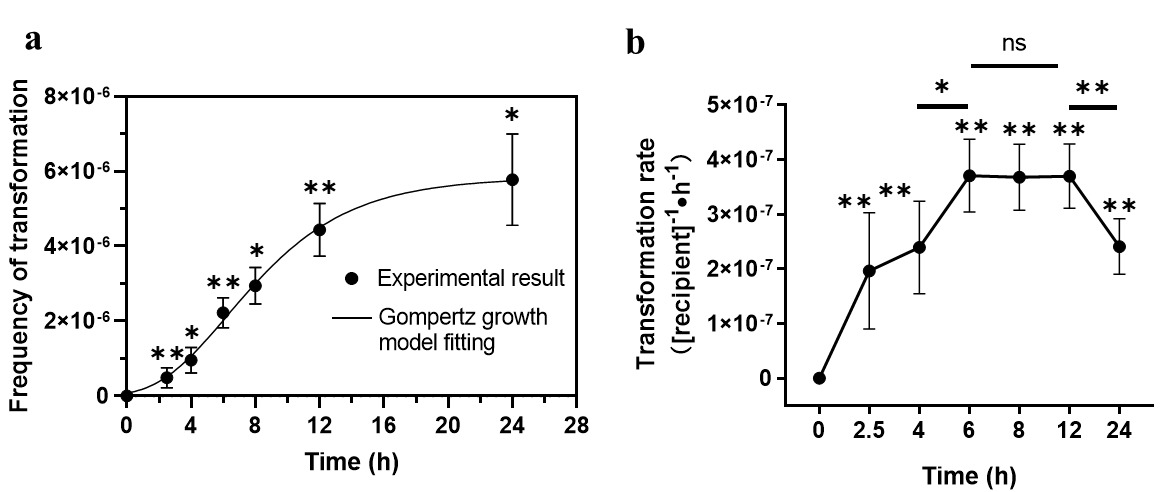


**Fig. S1. Effect of transformation time on the (a) frequency of transformation (*N* = 6) and (b) transformation rate (*N* = 6).** Transformation time has a significant effect on the frequency of transformation and transformation rate (ANOVA, *p* < 0.05). Significant differences between individual samples collected at different time intervals and the control (t = 0) as well as between the samples collected at 4 h, 6 h, 8 h, 12 h, and 24 h were tested with Independent-sample *t* test, ns *p* > 0.05, * *p* < 0.05, and ** *p* < 0.01.


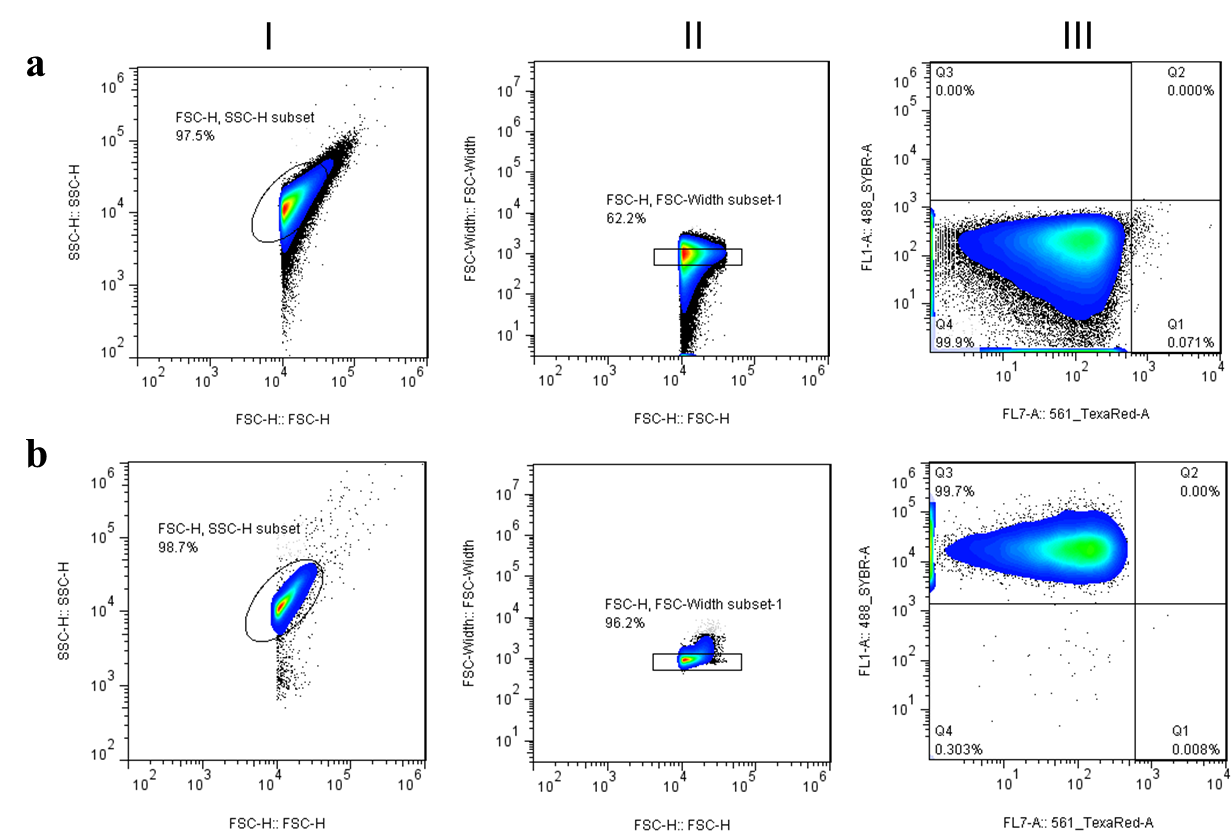


**Fig. S2. FACS sorting of transformant cells from a mixture initiated with faecal bacteria and *gfp*-pKJK5 plasmid.** Gate I sorts for bacterial size based on forward-H and side scatter-H; Gate II sorts for singlet based on forward-H and forward-W; and Gate III selects only those green cells (left corner). Panel (**a**) shows the results from faecal bacteria, while panel (b) shows the results from positive control (green transconjugants).


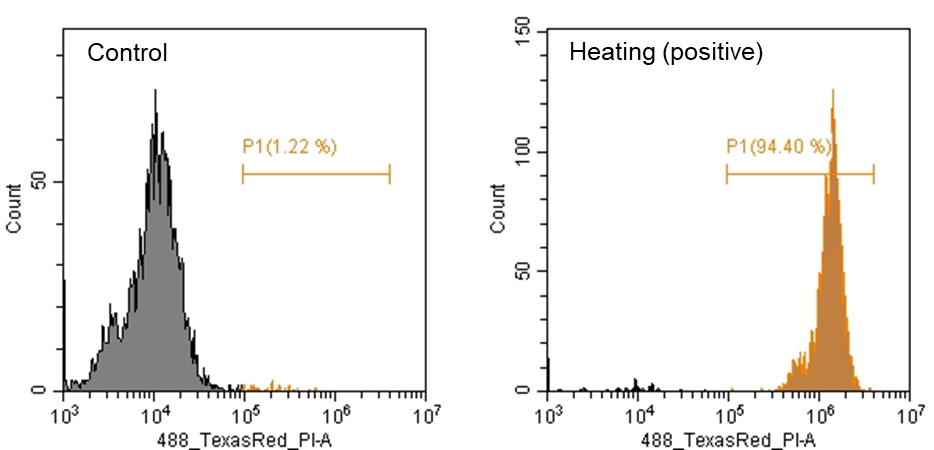


**Fig. S3. Histograms of flow cytometry analysis of the control (0 mg/L of sweeteners) and positive binding group (heated cells)**


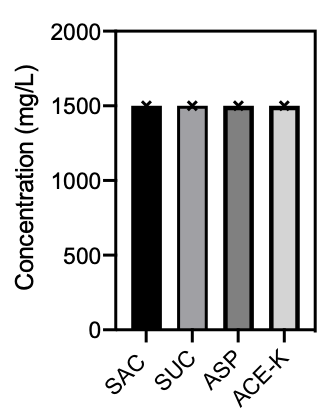


**Fig. S4.** **IC90 of the recipient** ***Acinetobacter baylyi* ADP1 against artificial sweeteners.** The marked “×” means that the values are higher than those of corresponding left Y axis values.

**Fig. S5. Monoculture growth curves. Each panel shows both monoculture growth curve data (marker) and best-fit growth model [4] (line).** Overnight culture of *A. baylyi* ADP1 was sub-cultured in fresh LB media and was used for growth assay under exposure to artificial sweeteners, at 30 ºC. All tests were conducted in triplicates. Estimated growth parameters such as max density, growth rate and lag time were summarized in Table S1.

**Fig. S6.** **Images of transformant plates showing the number of transformants induced by artificial sweeteners.** Images were taken by Uvitec UVIDOC HD6 Gel Doc System.

**Fig. S7.** **The number of recipient (*Acinetobacter baylyi* ADP1) treated with various concentrations of artificial sweeteners (*N* = 6).** Significant differences between individual artificial sweetener-treated groups (0.03, 0.3, 3, 30, 60 and 300 mg/L) and the control (0 mg/L of sweeteners) were tested with Independent-sample *t* test, * *p* < 0.05.

**
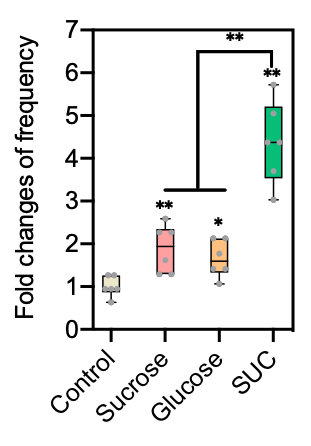
**

**Fig. S8. Comparisons of transformation frequency between sugars (sucrose and glucose)-treated groups and SUC-treated group (*N* = 6).** Each chemical was tested at 60 mg/L. Significant differences between individual sweetener-treated groups and the control (0 mg/L of sweeteners) were tested with Independent-sample *t* test, * *p* < 0.05 and ** *p* < 0.01.

**
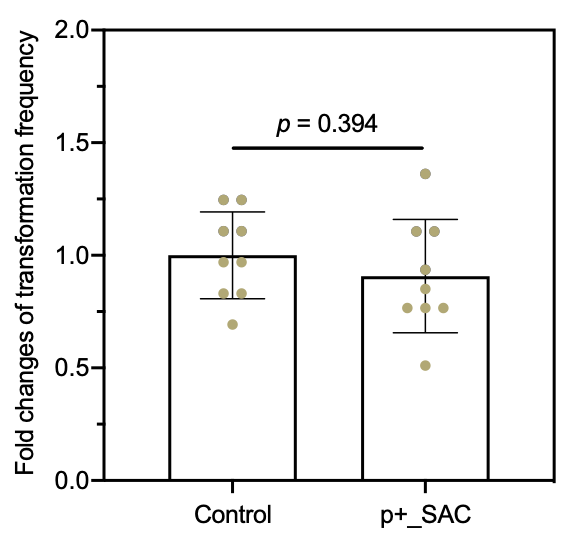
**

**Fig. S9. Effect of plasmid pre-exposure with SAC on transformation frequency.** The control is the spontaneous transformation group without addition of sweeteners; p+, means that the plasmid (p) pWH1266 was pre-exposed with 300 mg/L SAC for 2 h before added to bacterial suspension for transformation experiment.

**
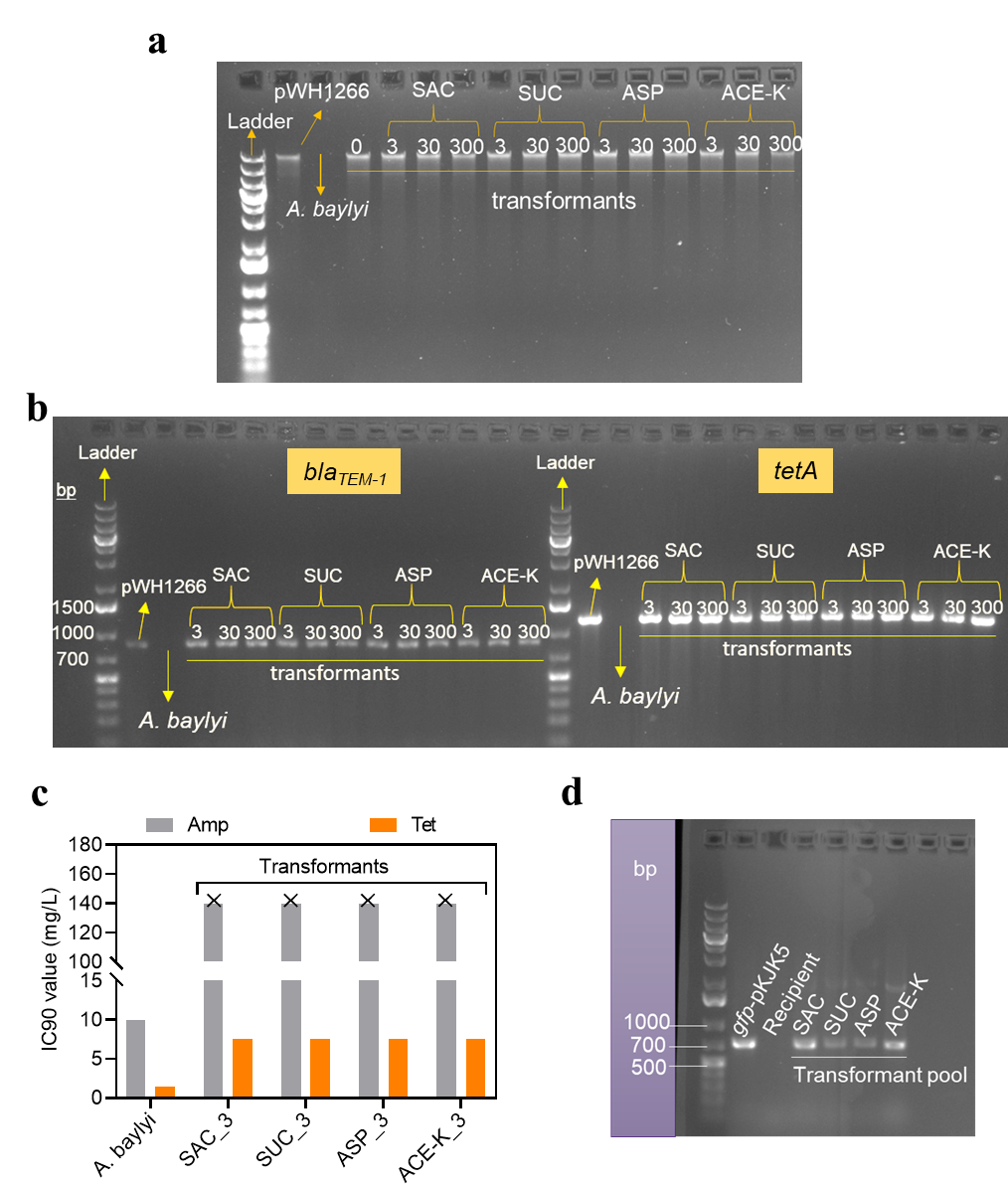
**

**Fig. S10. Verification of the successful transformation of pWH1266 plasmid in *A. baylyi* ADP1.** **a**, Plasmid extraction from the recipient and transformants and corresponding gel electrophoresis image. Transformants contain the plasmid band that the recipient does not have. **b**, PCR assays of ARGs (*bla_TEM-1_* and *tetA*) owned by the extracted plasmids and corresponding gel electrophoresis image. This further proves that transformants own the same plasmid to the pWH1266 plasmid. **c**, IC90 of the parent *A. baylyi* lacking pWH1266 plasmid and the transformants toward antibiotics (Amp and Tet), respectively (*N* = 3). The marked “**×**” in (**c**) means that the values are higher than those of corresponding left Y axis values. Transformants show higher resistance to the antibiotics than their parent bacterial cells. **d**, Gel electrophoresis image of *gfp* PCR assay before and after transformation experiment with faecal bacteria and *gfp*-encoded pKJK5 plasmid.

**Fig. S11. Effect of sugars (sucrose and glucose) on the transformation frequency of *gfp*-labelled pKJK5 plasmid in** **mice faecal bacteria.** The concentration of each sugar was 300 mg/L**.** Insignificant differences between individual sugar-treated groups and the control (0 mg/L of sugars) were observed (ns, *p* > 0.05).

**Fig. S12. Effect of sugars (sucrose and glucose) on cell envelope permeability of *A. baylyi* ADP1.** Each sample was analyzed in biological triplicate. Significant differences between individual sugar-treated groups and the control (0 mg/L of sugar) were tested with Independent-sample *t* test: ns means *p* value larger than 0.05.

**Fig. S13. Expression of efflux pump-related genes and proteins in *A. baylyi*** **ADP1** **after treated by 30 mg/L artificial sweeteners (SAC, SUC, ASP, ACE-K)**

**
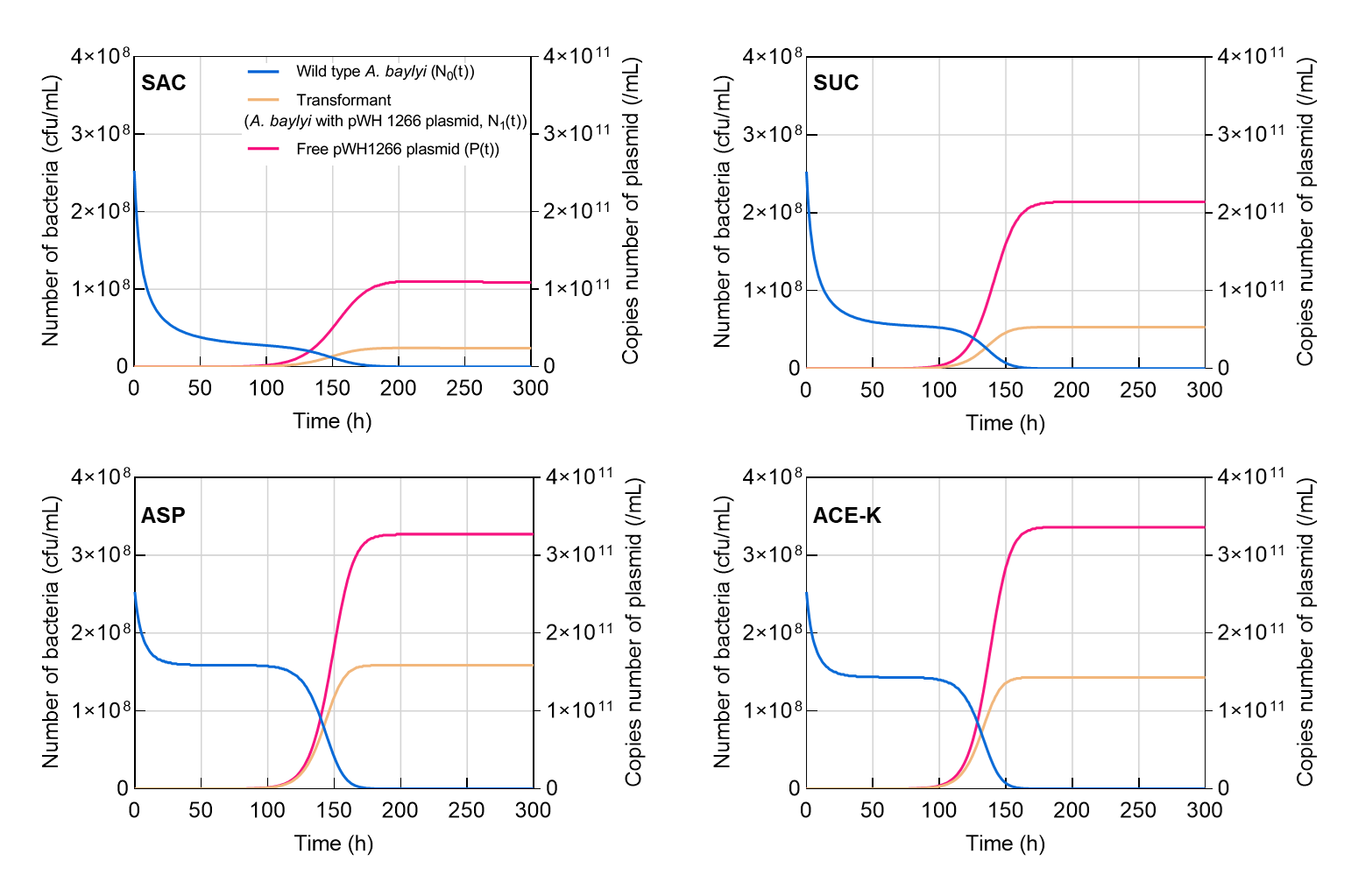
**

**Fig. S14.** **Simulated curves of the recipient number, transformant number and the number of plasmid copies in the presence of artificial sweeteners (30 mg/L)**

**Fig. S15. Concentrations of (a) sugars and (b) four artificial sweeteners in cell suspensions after 6-h incubation (*N* = 3).** The initial concentration of sucrose or glucose was 300 mg/L. The concentrations of glucose and sucrose were measured by Glucose (HK) Assay Kit (Sigma-Aldrich) and Sucrose Assay kit (Supelco), respectively. Initial concentrations of four sweeteners were selected at 3 mg/L. Each sample was run in biological triplicate. Significant differences between samples (the concentration was represented as C) from cell suspensions and the control (no cells; the concentration was represented as C_0_) were analysed using independent-sample *t* test and shown with ** *p* < 0.01.

**Artificial sweeteners induce insignificant reactive oxygen species production.** To demonstrate whether reactive oxygen species (ROS) could be induced to affect transformation process, we detected ROS concentration after exposed *Acinetobacter baylyi* ADP1 to four artificial sweeteners. Compared to the control, the detected ROS productions of the bacteria treated by four artificial sweeteners were not significantly increased (*p* = 0.057 ~ 1.000). The ROS production was even declined (*p* = 0.016) when treated with 300 mg/L SUC (Fig. S16a and b).

It has been reported that the fluorescein-based dye DCFDA (2’,7’-dichlorofluorescein diacetate) used for ROS detection can be more quickly oxidized in treated cells than in the untreated ones [5] and can be oxidized by the high-valence iron that is the by-product of Fenton reaction in cell [6, 7]. In this case, we confirmed whether the insignificant change of ROS production in the sweeteners treated groups was due to the oxidation of fluorescent reporter dye by high-valence iron of Fenton reaction rather than by generated ROS. Here, we used iron chelator 2,2’-dipyridyl (induced insignificant change of ROS production, Figs. S16c and S17) to sequester intracellular iron in order to avoid the preoxidation of DCFDA by Fenton reaction. Compared to the control (0 mg/L), the percent of ROS production significantly declined (*p* = 0.022) when added iron chelator (Fig. S16c). Although iron chelator significantly decreased (*p* = 0.015) ROS production in 300 mg/L SAC-treated group, it caused no significant (*p* = 0.051 ~ 0.693) decrease in other treated groups.

To further explore the formation of intracellular ROS, we next sought to quench the generated ROS via Fenton reaction by adding thiourea to artificial sweetener-treated cell suspensions. We observed that in the control group (0 mg/L), thiourea significantly (*p* = 0.012) decreased the ROS production (Fig. S16d) to the same extent (0.15% ROS production) that iron chelator did. This result was also found when added thiourea to the groups treated by 300 mg/L of all artificial sweeteners.

In this study, we also extracted and sequenced proteins from the mixture containing plasmid and the recipient. Consistent with the results of ROS production, all artificial sweeteners did not significantly regulate the expressions of ROS-related proteins (Supplementary Data File 1). Altogether, artificial sweeteners induced insignificant increase of ROS production as a result of lower intensity of ROS-generating Fenton reaction rather than the preoxidation of fluorescent dye.

**Fig. S16.** **Artificial sweeteners did not induce significant ROS production of *Acinetobacter baylyi* ADP1. (a)**, Percent of ROS production induced by SAC and SUC. **(b)**, Percent of ROS production induced by ASP and ACE-K. **(c)**, Generation of ROS (%) with or without iron chelator 2,2’-dipydyl. **(d)**, Generation of ROS (%) with or without ROS scavenger thiourea (TU). Significant differences between individual artificial sweetener-treated groups and the control (0 mg/L of sweeteners) were tested with Independent-sample *t* test, * *p* < 0.05 and ** *p* < 0.01.


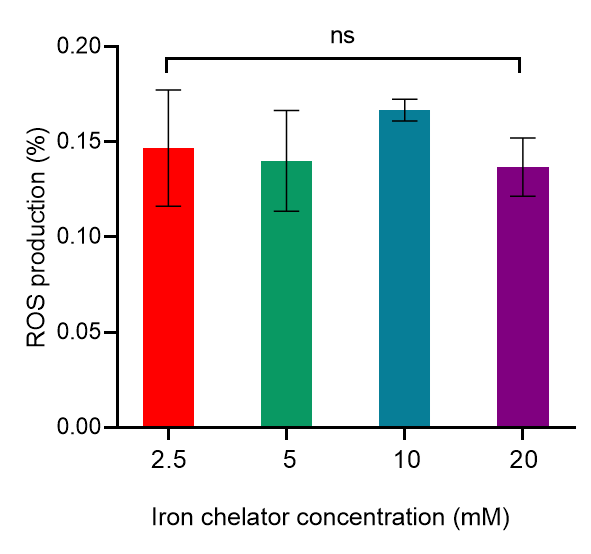


**Fig. S17.** **Effects of different iron chelator concentrations on the percent of ROS production in *Acinetobacter baylyi* ADP1.** No significant difference was observed in terms of *p* values larger than 0.05.

**Table S1. Physical-chemical properties of four artificial sweeteners**

| Artificial Sweeteners | Chemical structure | Formula | Molecular Weight | Water solubility^a^ | Sweetness^b^ |
| --- | --- | --- | --- | --- | --- |
| Saccharine | 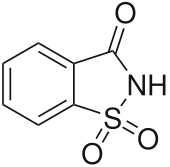 | C_7_H_5_NO_3_S | 183.18 g·mol^−1^ | 789.2 | ~ 300 |
| Sucralose | 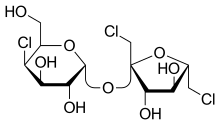 | C_12_H_19_Cl_3_O_8_ | 397.64 g/mol | 2.75 × 10^4^ | ~ 600 |
| Aspartame | 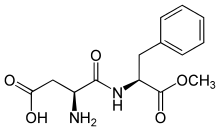 | C_14_H_18_N_2_O_5_ | 294.31 g·mol^−1^ | 564.7 | > 200 |
| Acesulfame potassium | 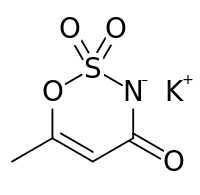 | C_4_H_4_KNO_4_S | 201.242 g·mol^−1^ | 9.1 × 10^5^ | 200 |

^a^ Measured at 25 ºC (mg/L) [8].

^b^ Compared to sucrose. Data are cited from Food Standards in Australia-New Zealand (<https://www.foodstandards.gov.au/consumer/additives/Pages/Sweeteners.aspx>).

**Table S2. PCR primers used in this study**

| Primer | Amplicon size | Primer length | | Sequence (5′ to 3′) | Annealing temperature |
| --- | --- | --- | --- | --- | --- |
| *bla_TEM-1_* Long Forward | 861 bp | 24 bp | TTACCAATGCTTAATCAGTGAGGC | | 51.4 ºC |
| *bla_TEM-1_* Long Reverse |  | 25 bp | ATGAGTATTCAACATTTCCGTGTCG | |  |
| *tetA* Long Forward | 1200 bp | 25 bp | CGTGTATGAAATCTAACAATGCGCT | | 51.9 ºC |
| *tetA* Long Reverse |  | 19 bp | CCATTCAGGTCGAGGTGGC | |  |
| *gfp* Forward |  |  | ATATAGCATGCGTAAAGGAGAAGAACTTTTCA | | 51 ºC |
| *gfp* Reverse |  |  | CTCTCAAGCTTATTTGTATAGTTCATCCATGC | |  |

**Table S3. Benchmark points of the scale factor**

| Sequence number | 1 | 2 | 3 | 4 | 5 | 6 | 7 | 8 | 9 |
| --- | --- | --- | --- | --- | --- | --- | --- | --- | --- |
| Benchmark point of scale factor | 0.05 | 0.1 | 0.2 | 0.5 | 1 | 2 | 5 | 10 | 20 |

**Table S4. Variation range of *K_μ_***

| A | B | C | D | E | F | G | H |
| --- | --- | --- | --- | --- | --- | --- | --- |
| 0.05-0.1 | 0.1-0.2 | 0.2-0.5 | 0.5-1 | 1-2 | 2-5 | 5-10 | 10-20 |

**Table S5. Variation range of** ***K_d_***

| *a* | *b* | *c* | *d* | *e* | *f* | *g* | *h* |
| --- | --- | --- | --- | --- | --- | --- | --- |
| 0.05-0.1 | 0.1-0.2 | 0.2-0.5 | 0.5-1 | 1-2 | 2-5 | 5-10 | 10-20 |

**Table S6. Feasible subranges of the model calibration with two decision variables**

| *Ωd*  *Ωμ* | *a* | *b* | *c* | *d* | *e* | *f* | *g* | *h* |
| --- | --- | --- | --- | --- | --- | --- | --- | --- |
| *A* | *A-a* | *A-b* | *A-c* | *A-d* | *A-e* | *A-f* | *A-g* | *A-h* |
| *B* | *B-a* | *B-b* | *B-c* | *B-d* | *B-e* | *B-f* | *B-g* | *B-h* |
| *C* | *C-a* | *C-b* | *C-c* | *C-d* | *C-e* | *C-f* | *C-g* | *C-h* |
| *D* | *D-a* | *D-b* | *D-c* | *D-d* | *D-e* | *D-f* | *D-g* | *D-h* |
| *E* | *E-a* | *E-b* | *E-c* | *E-d* | *E-e* | *E-f* | *E-g* | *E-h* |
| *F* | *F-a* | *F-b* | *F-c* | *F-d* | *F-e* | *F-f* | *F-g* | *F-h* |
| *G* | *G-a* | *G-b* | *G-c* | *G-d* | *G-e* | *G-f* | *G-g* | *G-h* |
| *H* | *H-a* | *H-b* | *H-c* | *H-d* | *H-e* | *H-f* | *H-g* | *H-h* |

**Table S7. Summary of parameters involved in calibration and benchmark point (*L_μ_, L_d_*) determination**

| Parameter | Description |
| --- | --- |
| *LB_μ_* | Lower bound of *K_μ_* |
| *LB_d_* | Lower bound of *K_d_* |
| *Ω_μ_* | Variation range of *K_μ_* |
| *L_𝜇_* | Benchmark point of *K_μ_*, with the minimum value of LS function |
| *A~H* | Symbols of various Ω_μ_ |
| *R_𝜇_* | Benchmark point of scale factor for transformation frequency |
| Φ | Set of benchmark points for scale factor |
| *UB_μ_* | Upper bound of *K_μ_* |
| *UB_d_* | Upper bound of *K_d_* |
| *Ω_d_* | Variation range of *K_d_* |
| *L_d_* | Benchmark point of *K_d_*, with the minimum value of LS function |
| *a~h* | Symbols of various Ω_d_ |
| *R_d_* | Benchmark point of scale factor for death rate |

**Table S8. The optimal *L_μ_* and *L_d_* values, search ranges *Ω_μ_-Ω_d_ of K_μ_ and K_d_,* and the associated LB and UB values under different categories**

| Category | *L_μ_* | *L_d_* | *Ωμ-Ωd* | LB_μ_ | UB_μ_ | LB_d_ | UB_d_ |
| --- | --- | --- | --- | --- | --- | --- | --- |
| Control | 2 | 5 | AB de | 0.05 | 0.2 | 0.5 | 2 |
| SAC | 3 | 6 | BC ef | 0.1 | 0.5 | 1 | 5 |
| SUC | 3 | 6 | BC ef | 0.1 | 0.5 | 1 | 5 |
| ASP | 2 | 5 | AB de | 0.05 | 0.3 | 0.5 | 2 |
| ACE-K | 2 | 5 | AB de | 0.05 | 0.3 | 0.5 | 2 |

**Table S9. The settings in Optimization toolbox 7.3 during the Genetic Algorithm**

| Population | Type | Double Vector |
| --- | --- | --- |
|  | Size | 50 |
|  | Creation Function | Constraint Dependent |
| Fitness Scaling | | Rank |
| Selection | | Stochastic Uniform |
| Reproduction | Elite Count | 0.05*Size |
|  | Crossover Fraction | 0.8 |
| Mutation | | Constraint Dependent |
| Crossover | | Constraint Dependent |
| Migration | Direction | Forward |
|  | Fraction | 0.2 |
|  | Interval | 20 |

**Table S10. Estimated growth parameters of *A. baylyi* ADP1 under exposure to artificial sweeteners**

| Artificial sweeteners | Concentration, mg/L | Max density (*K*) | Growth rate (h^-1^) | Lag time (h) |
| --- | --- | --- | --- | --- |
| SAC | 0 | 0.393 (0.386, 0.399) | 0.887 (0.818, 0.965) | 1.127 (1.037, 1.222) |
|  | 60 | 0.389 (0.385, 0.393) | 0.769 (0.818, 0.965) | 1.301 (1.224, 1.382) |
|  | 300 | 0.370 (0.364, 0.376) | 0.723 (0.694, 0.754) | 1.383 (1.327, 1.442) |
| SUC | 0 | 0.393 (0.386, 0.399) | 0.887 (0.818, 0.965) | 1.127 (1.037, 1.222) |
|  | 60 | 0.399 (0.396, 0.403) | 0.675 (0.653, 0.697) | 1.482 (1.434, 1.532) |
|  | 300 | 0.386 (0.380, 0.392) | 0.607 (0.582, 0.634) | 1.647 (1.578, 1.719) |
| ASP | 0 | 0.393 (0.386, 0.399) | 0.887 (0.818, 0.965) | 1.127 (1.037, 1.222) |
|  | 60 | 0.399 (0.394, 0.404) | 0.683 (0.652, 0.715) | 1.464 (1.398, 1.533) |
|  | 300 | 0.368 (0.363, 0.374) | 0.609 (0.580, 0.638) | 1.643 (1.566, 1.724) |
| ACE-K | 0 | 0.393 (0.386, 0.399) | 0.887 (0.818, 0.965) | 1.127 (1.037, 1.222) |
|  | 60 | 0.388 (0.383, 0.393) | 0.648 (0.618, 0.679) | 1.544 (1.474, 1.617) |
|  | 300 | 0.339 (0.334, 0.344) | 0.686 (0.651, 0.723) | 1.458 (1.383, 1.536) |

Parentheses provide 95% confidence intervals. Densities are OD_600_; growth rate and lag time are in hour.

**Table S11. Simulation and calculation results of natural transformation under exposure to artificial sweeteners**

| Treatment | *K_μ_** | Amplify factor of *K_μ_** (Fold change) | *K_d_** | Amplify factor of *K_d_** (Fold change) | Stability time (h) |
| --- | --- | --- | --- | --- | --- |
| Control | 0.125 | 1 | 0.995 | 1 | 215 |
| SAC | 0.291 | 2.328 | 1.758 | 1.767 | 183 |
| SUC | 0.239 | 1.912 | 1.459 | 1.466 | 159 |
| ASP | 0.191 | 1.528 | 0.747 | 0.751 | 164 |
| ACE-K | 0.202 | 1.616 | 0.853 | 0.857 | 152 |
| Combination | 1.374 | 10.99 | 1.659 | 1.667 | 44 |

**References**

1. Maeusli M, Lee B, Miller S, Reyna Z, Lu P, Yan J, et al. Horizontal gene transfer of antibiotic resistance from *Acinetobacter baylyi* to *Escherichia coli* on lettuce and subsequent antibiotic resistance transmission to the gut microbiome*.* Msphere. 2020;5.

2. Neil K, Allard N, Grenier F, Burrus V,Rodrigue S. Highly efficient gene transfer in the mouse gut microbiota is enabled by the IncI 2 conjugative plasmid TP114*.* Commun Biol. 2020;3:1-9.

3. Yu Z, Wang Y, Lu J, Bond PL,Guo J. Nonnutritive sweeteners can promote the dissemination of antibiotic resistance through conjugative gene transfer*.* ISME J. 2021:1-14.

4. Ram Y, Dellus-Gur E, Bibi M, Karkare K, Obolski U, Feldman MW, et al. Predicting microbial growth in a mixed culture from growth curve data*.* Proc Natl Acad Sci USA. 2019;116:14698-14707.

5. Setsukinai KI, Urano Y, Kakinuma K, Majima HJ,Nagano T. Development of novel fluorescence probes that can reliably detect reactive oxygen species and distinguish specific species*.* J Biol Chem. 2003;278:3170-3175.

6. Liu Y,Imlay JA. Cell death from antibiotics without the involvement of reactive oxygen species*.* Science. 2013;339:1210-1213.

7. Kohanski MA, Dwyer DJ, Hayete B, Lawrence CA,Collins JJ. A common mechanism of cellular death induced by bactericidal antibiotics*.* Cell. 2007;130:797-810.

8. Subedi B,Kannan K. Fate of artificial sweeteners in wastewater treatment plants in New York State, U.S.A*.* Environ Sci Technol. 2014;48:13668-74.
